# Secretory Phosphatases Deficient Mutant of *Mycobacterium tuberculosis* Imparts Protection at the Primary Site of Infection in Guinea Pigs

**DOI:** 10.1371/journal.pone.0077930

**Published:** 2013-10-18

**Authors:** Priyanka Chauhan, P. Vineel Reddy, Ramandeep Singh, Neetika Jaisinghani, Sheetal Gandotra, Anil K. Tyagi

**Affiliations:** 1 Department of Biochemistry, University of Delhi South Campus, New Delhi, India; 2 CSIR-Institute of Genomics and Integrative Biology, New Delhi, India; Hopital Raymond Poincare - Universite Versailles St. Quentin, France

## Abstract

**Background:**

The failure of *Mycobacterium bovis* Bacille Calmette-Guérin to impart satisfactory protection against adult pulmonary tuberculosis has necessitated the development of more effective TB vaccines. The assumption that the vaccine strain should be antigenically as similar as possible to the disease causing pathogen has led to the evaluation of *M.tuberculosis* mutants as candidate tuberculosis vaccines.

**Methods/Principal Findings:**

In this study, we have generated a mutant of *M.tuberculosis* (Mtb∆*mms*) by disrupting 3 virulence genes encoding a mycobacterial secretory acid phosphatase (*sapM*) and two phosphotyrosine protein phosphatases (*mptpA* and *mptpB*) and have evaluated its protective efficacy in guinea pigs. We observed that Mtb∆*mms* was highly attenuated in THP-1 macrophages. Moreover, no bacilli were recovered from the lungs and spleens of guinea pigs after 10 weeks of Mtb∆*mms* inoculation, although, initially, the mutant exhibited some growth in the spleens. Subsequently, when Mtb∆*mms* was evaluated for its protective efficacy, we observed that similar to BCG vaccination, Mtb∆*mms* exhibited a significantly reduced CFU in the lungs of guinea pigs when compared with the unvaccinated animals at 4 weeks after challenge. In addition, our observations at 12 weeks post challenge demonstrated that Mtb∆*mms* exhibited a more sustainable and superior protection in lungs as compared to BCG. However, the mutant failed to control the hematogenous spread as the splenic bacillary load between Mtb∆*mms* vaccinated and sham immunized animals was not significantly different. The gross pathological observations and histopathological observations corroborated the bacterial findings. Inspite of disruption of phosphatase genes in MtbΔ*mms*, the lipid profiles of *M.tuberculosis* and MtbΔ*mms* were identical indicating thereby that the phenotype of the mutant was ascribed to the loss of phosphatase genes and the influence was not related to any alteration in the lipid composition.

**Conclusions/Significance:**

This study highlights the importance of *M.tuberculosis* mutants in imparting protection against pulmonary TB.

## Introduction

 Tuberculosis (TB) continues to intimidate human race unabashedly and remains a major cause of morbidity and mortality throughout the world [1,2]. Every week, more than 150,000 individuals develop TB and ~30,000 human lives are lost globally due to this dreaded disease. The lethal liaison between TB and HIV infections and the emergence of various forms of drug resistant *M.tuberculosis* strains have made the situation even more precarious [3,4]. Although, the current vaccine, *Mycobacterium bovis* Bacille Calmette-Guérin (BCG) does provide protection against childhood TB especially TB meningitis, it is ineffective in providing consistent protection against the disease in adults and older people [5]. Under the best of the circumstances, it has provided 80% protection, which generally has been to the tune of 40-60% on an average. Therefore, the need to develop a superior TB vaccine than BCG cannot be over-emphasized.

 The purpose of an effective live vaccine would be best served if the vaccine strain is antigenically as similar as possible to the disease-causing pathogen in order for it to generate the host immune responses that mimic natural infection [6]. Comparative genomic studies have revealed that BCG, in comparison to *M.tuberculosis*, lacks 16 defined regions (RD1-16) comprising of ~150 genes, some of which are known to encode potential antigenic determinants that could increase the immunogenicity of a vaccine [7,8]. This makes the use of attenuated *M.tuberculosis* strains rather than BCG, for the generation of appropriate immune responses, an attractive idea [5,9,10]. Several *M.tuberculosis* mutants have been evaluated in animal models and have resulted in varying degrees of success in imparting protection against TB when compared with BCG [11–15]. Immunization of mice with the ∆RD1∆*panCD* mutant of *M.tuberculosis* (an attenuated *M.tuberculosis* RD1 knockout and pantothenate auxotroph) resulted in 1-2 log_10_ CFU lower bacillary loads in the spleens, lungs and liver when compared with the BCG. However, in bull calves, no histopathological differences were observed in the lung and lymph nodes of ∆RD1∆*panCD* vaccinees when compared with the unvaccinated controls [14,15]. Similarly, mice vaccinated with ∆*secA2* mutant (sec *A2* deletion mutant of *M.tuberculosis*) exhibited significantly lower pulmonary and splenic CFU when compared with the BCG vaccinated group, however, the same vaccine performed as well as BCG in guinea pigs [11]. In contrast to these observations, Martin et al. demonstrated similar level of protection exhibited by SO2 strain (*phoP* deletion mutant of *M.tuberculosis*) in mice, although guinea pigs vaccinated with SO2 exhibited significantly increased survival time when compared with BCG [12]. The variable results shown by the candidate vaccines and the fact that none of the current candidates has successfully made through the clinical trials reinforce the importance of keeping the pipeline full with the new candidates [16].

 Among the secretory proteins of *M.tuberculosis*, three phosphatases, namely, mycobacterial secretory acid phosphatase (SapM) and two phosphotyrosine protein phosphatases (MptpA and MptpB) have been shown to contribute to its pathogenicity [17–20]. SapM dephosphorylates phosphatidylinositol 3-phosphate (PI3P), a membrane trafficking regulatory lipid, resulting in the arrest of phagosome maturation [19]. In addition, in a study by Festjens et al, disruption of *sapM* locus in BCG improved its protective efficacy as a vaccine against TB [21]. The increased efficacy of the vaccine was accredited to the efficient activation and recruitment of dendritic cells to the draining lymph nodes in the absence of SapM, thus allowing successful antigen presentation and activation of the adaptive immunity by dendritic cells [21]. A recent study showed that the *fbpA*/*sapM*  double mutant of *M.tuberculosis* was attenuated for growth and more immunogenic in macrophages as compared to *M.tuberculosis* [22].

 MptpA has been demonstrated to block phagosome-lysosome fusion by inhibiting V-ATPase trafficking to the mycobacterial phagosome [23–25]. It has been reported that *mptpA* mutant of *M.tuberculosis* was impaired for survival/growth in THP-1 macrophages and phagosomes harboring the mutant strain exhibited increased phagosome-lysosome fusion [23]. It has been previously reported that *M.tuberculosis* devoid of MptpB activity was impaired for survival in IFN-γ activated macrophages and in guinea pigs [26]. In another study, it was shown that MptpB inhibits ERK ½, p38 signaling pathways and caspase 3 activity, thus subverting the host immune response to infection [27]. The importance of MptpB in the intracellular survival of *M.tuberculosis* was also demonstrated in a study in which specific inhibitors against MptpB were shown to inhibit mycobacterial survival within murine macrophages [17,27].

 In this study, by deleting the function of three virulence genes, namely, *mptpA* (*Rv2234*)*, mptpB* (*Rv0153c*) and *sapM* (*Rv3310*), we have developed the mutant Mtb*∆mms* and evaluated its protective efficacy in guinea pig model of experimental tuberculosis. 

## Materials and Methods

### Bacterial strains and growth conditions

 The bacterial strains and plasmids used in this study are listed in Table 1. *M. bovis* BCG (Danish strain) was obtained from BCG laboratories, Chennai, India. *M.tuberculosis* H37Rv (ATCC No. 25618) used for challenge was procured from Dr. J. S. Tyagi, AIIMS, New Delhi, India. Mycobacterial strains were grown to mid-log phase in MB7H9 medium supplemented with 1X albumin-dextrose-catalase (ADC), 0.5% glycerol and 0.05% Tween 80. PBS stocks were prepared and stored at -80°C till further use. The CFU of stocks was enumerated by plating appropriate dilutions in duplicates on MB7H11 agar supplemented with 1X oleic acid-albumin-dextrose-catalase (OADC) and 0.5% glycerol. *E.coli* strains, XL-1 Blue (Stratagene) and HB101 (Life Technologies) were used for cloning purposes. Kanamycin and Chloramphenicol were used at 25 μg/ml and 30 μg/ml, respectively. Hygromycin was used at 50 μg/ml for *M.tuberculosis* and at 150 μg/ml for *E.coli*. 

**Table 1:** Bacterial strains, plasmids, cell line and primers used in this study.

**Strains/Plasmids/Cell line/Primers**	**Description**	**Reference**
**Strains**		
*E.coli* XL-1 Blue	*endA*1 *gyrA*96 (*nalR*) *thi*-1 *recA*1 *relA*1 *lac glnV*44 F' [::Tn10 *proAB* + *lacIq* Δ (*lacZ*) M15] *hsdR17* (rK- mK+)	Stratagene, Heidelberg, Germany
*E.coli* HB101	F-(*gpt-proA*) 62 *leuB*6 *glnV*44 *ara*-14 *galK*2 *lacY*1 (*mcrC-mrr*) *rpsL*20 (Strr) *xyl*-5 *mtl*-1 *recA*13	Life Technologies, CA, USA
Mtb*∆mptpB*	*M.tuberculosis* Erdman *mptpB* mutant	[26]
Mtb∆*mptpB*∆*mptpA*	*M.tuberculosis mptpB* and *mptpA* mutant	This study
MtbΔ*mms*	*M.tuberculosis mptpA*, *mptpB* and *sapM* mutant	This Study
*M.tuberculosis* H37Rv (ATCC No. 25618)	Virulent strain of *M.tuberculosis*	Dr. J. S. Tyagi, AIIMS, New Delhi, India
*M.bovis* BCG Danish	Vaccine strain against tuberculosis	BCG laboratories, Chennai, India
**Plasmids**		
pLIT∆A	pLitmus vector carrying the amplicons of *mptpA* with a deletion of 169 bp from the central region of ORF	This study
pLIT∆AK	pLitmus vector with kanamycin resistance gene cassette flanked with *mptpA* amplicons	This study
pYUB∆*SapM*	Cloning vector with Hygromycin resistance gene cassette flanked with *sapM* amplicons	unpublished data
pYUB.*CATtrrn*∆*sapM*	Cloning vector with Chloramphenicol resistance gene under mycobacterial trrn promoter flanked with *sapM* amplicons	This study
pVR1	A derivative of pSD5 containing Chloramphenicol resistance gene under mycobacterial *trrn* promoter	[51]
**Cell line**		
THP-1	The Human acute monocytic leukemia cell line	NCCS, Pune, India
**Primers**		
F-*mptpA*	5′-gtgtctgatccgctgcacgtcacattc-3′	This study
R-*mptpA*	5′-tcaactcggtccgttccgcgcgagac-3′	This study
F-*sapM*	5′-atgctccgcggaatccaggctc-3′	This study
R-*sapM*	5′-ctagtcgccccaaatatcgg-3′	This study

### Generation of Mtb*∆mms* Mutant of *M.tuberculosis*

To generate Mtb∆*mms* mutant of *M.tuberculosis*, a portion of *mptpA* and *sapM* was deleted in the genome of Mtb∆*mptpB* [26] and replaced with Kanamycin resistance cassette and Chloramphenicol resistance cassette, respectively. For the generation of MtbΔ*mptpB*Δ*mptpA* double gene mutant, primers were designed to amplify (i) 156 bp of 5′ proximal end of *mptpA* along with 1135 bp of immediate upstream region of *mptpA* (amplicon I) and (ii) 167 bp of 3′ distal end of *mptpA* along with 1240 bp of immediate downstream region of *mptpA* (amplicon II). The amplicons I and II were PCR amplified and cloned into the vector pLitmus-38 (New England Biolabs) to generate the vector pLITΔA (with a deletion of 169 bp from the central region of *mptpA* ORF). The Kanamycin resistance gene was excised out from pSD5 as an *Nhe*I-*Bst*EII fragment, end-repaired and cloned into *Nde*I digested, end-repaired pLITΔA to generate pLIT38ΔAK. The vector pLIT38ΔAK was pretreated with alkali [28] and 2 μg of DNA was electroporated into Mtb∆*mptpB* electrocompetent cells to generate a double gene mutant, namely, Mtb∆*mptpB*∆*mptpA*. 

For the generation of Mtb*∆mms* mutant of *M.tuberculosis*, *sapM* was disrupted in Mtb∆*mptpB*∆*mptpA*. For this, we employed a modified pYUB∆*sapM* vector generated in our laboratory (Table 1) by replacing hygromycin resistance cassette in pYUB∆*sapM* with the Chloramphenicol resistance gene expressed under the mycobacterial *trrn* promoter, *CATtrrn*. Briefly, primers were designed to amplify (i) ~700 bp amplicon comprising of ~200 bp of 5′ proximal end of *sapM* and ~500 bp of immediate upstream region of *sapM* (amplicon I) and (ii) ~700 bp amplicon comprising of ~200 bp of 3′ distal end of *sapM* and ~500 bp of immediate downstream region of *sapM* (amplicon II). Both amplicons were cloned into pYUB854 flanking the hygromycin resistance cassette to generate pYUB∆*sapM*. The hygromycin resistance cassette in pYUB∆*sapM* was replaced with *CATtrrn* (The *CATtrrn* was PCR amplified from pVR1, Table 1) resulting in pYUB.*CATtrrn*∆*sapM*. A linear Allelic Exchange Substrate (AES), Δ*sapM*::*CATtrrn* was then excised out as *Kpn*I/*Spe*I fragement and electroporated into MtbΔ*mptpB*Δ*mptpA* to generate the mutant which was designated as MtbΔ*mms* (*mptpA*, *mptpB* and *sapM*).

### Confirmation of deletion of *mptpA* and *sapM* by Southern hybridization

 To confirm the deletion of *mptpA* in MtbΔ*mptpB*Δ*mptpA*, the genomic DNA was isolated from the parental strain (MtbΔ*mptpB*) and the mutant strain (MtbΔ*mptpB*Δ*mptpA*) followed by the digestion of 2 µg of DNA with *Pvu*II. The deletion of *sapM* in MtbΔ*mms* was confirmed by isolating the genomic DNA from MtbΔ*mptpB*Δ*mptpA* and MtbΔ*mms*, followed by digestion of 2 μg of DNA with *Pst*I. DNA was electrophoresed through 1% agarose gel followed by depurination, denaturation and neutralization of DNA within the agarose gel. DNA was then transferred onto positively charged nylon membrane by capillary transfer overnight and immobilized by UV radiation. 200 bp region at 5′ termini of *mptpA* and *sapM* were amplified for the generation of probe. The probe labeling, subsequent pre-hybridization, hybridization and detection were performed as described in the DIG High Prime DNA Labeling and Detection Starter Kit II (Roche Applied Science, IN, USA).

### Lipid profile analysis

#### Isolation of mycolic acids

Mycolic acids were extracted from *M.tuberculosis* as well as MtbΔ*mms* as described previously [29]. Briefly, mycobacterial strains were grown in 50 ml of MB7H9 supplemented with 1X ADC to an A_600_ of 1.0. The culture was harvested, heat killed (95°C for 1 hr) and then saponified with 6 ml of 20% tetrabutylammonium hydroxide at 100°C, overnight, to hydrolyze the mycolic acids from the cell wall. Free mycolic acids so generated were methylated by adding 1:1 dichloromethane methyleuchlorid and 300 μl of methyl iodide to form mycolic acid methyl esters. Upon phase separation, the lower organic layer was collected, dried and re-suspended in diethyl ether (3 ml). This lipid suspension was centrifuged at 2500 rpm for 2-3 min and the supernatant was collected and dried. The crystals thus formed, were suspended in 900 μl of a mixture of toluene and acetonitrile (2: 1). The solution was transferred to a microcentrifuge tube followed by addition of 600 μl of acetonitrile to the suspension. The suspension was then frozen at -20°C overnight. The solution was centrifuged at 12000 rpm at 4°C for 15 min. Finally, the pellet was suspended in 500 μl of diethyl ether and transferred to a small glass tube and evaporated with liquid nitrogen. The equivalent amount of mycolic acids extracted from *M.tuberculosis* as well as MtbΔ*mms*, suspended in diethyl ether were spotted on a thin layer chromatography (TLC) plate (Merck, TLC Aluminium sheets silica gel 60), chromatographed in hexane: ethylacetate (95: 5, v/v) seven times and visualized by staining with 20% sulphuric acid in ethanol followed by charring.

#### Extraction of polar and apolar lipids

Mycobacterial lipids were extracted as described previously [30]. Briefly, 50 ml of mycobacterial cultures, grown in MB7H9 supplemented with 1X ADC, were harvested at an A_600nm_ of 1.0 and heat killed (95°C for 1 hr). Apolar lipids were extracted by adding 2 ml of methanolic solution of 0.3% sodium chloride and 1 ml of petroleum ether (60-80°C) to the cell pellet. The cell suspension was mixed end-over-end for 30 min followed by centrifugation at 2500 rpm for 10 min. The upper layer consisting of apolar lipids was collected in a separate vial and 1 ml of petroleum ether was added to the lower layer, vortexed and mixed end-over-end for 15 min. The cell suspension was again centrifuged to recollect the upper layer. The upper layers comprising of apolar lipids were pooled and dried at 60°C. 

 Further, the polar lipids were extracted by adding 2.3 ml of chloroform: methanol: 0.3% sodium chloride (90: 100: 30, v/v/v) to the bottom layer. The cell suspension was mixed end- over-end for 60 min followed by centrifugation at 2500 rpm for 10 min. Polar lipids, present in the supernatant fraction, were collected and the pellet was further treated twice with 750 μl of chloroform: methanol: 0.3% sodium chloride (50: 100: 40, v/v/v), to obtain all polar lipids. The supernatants from these three extractions were pooled and further extracted with 1.3 ml of chloroform and 1.3 ml of 0.3% sodium chloride. The lower layer comprising of polar lipids was collected into a fresh glass tube and dried at 60°C. Equivalent amounts of polar and aploar lipids suspended in chloroform: methanol (2: 1, v/v) from both *M.tuberculosis* and MtbΔ*mms* strains were then spotted on TLC plates and analysed for different lipid fractions by using different solvent system as described in Table S1. TLC plates were developed by dipping in 10% phosphomolybdate or spraying with 2% orcinol in 10% sulphuric acid (for solvent C) followed by charring.

 For the detection of trehalose monomycolate (TMM), trehalose dimycolate (TDM) and sulfolipids (SL), 5 μCi of 14C-acetate was added to 10 ml of log phase culture of both *M.tuberculosis* and MtbΔ*mms* strains, separately. Cultures were then harvested after 18 hrs of radioactive pulse and apolar lipids were extracted from a methanolic solution of 0.3% sodium chloride and petroleum ether as described above. The organic phase was suspended in chloroform: methanol (2: 1, v/v). Approximately, 25,000 counts from the samples belonging to each strain were spotted on the TLC plate followed by chromatography in the appropriate solvents (Table S1). The lipids were visualized with a Typhoon FLA 700 Phosphorimager.

### Comparison of the growth of MtbΔ*mms* and the parental strain in human macrophages

 Human monocytic THP-1 cells were cultured in complete RPMI-GlutaMAX^TM^ medium [containing 10% heat inactivated FBS and 1% antibiotic-antimycotic mix] (GIBCO Grand Island, NY, USA) and were differentiated to macrophages by the addition of 30 nM Phorbol 12-myristate 13-acetate (PMA, Sigma) for 16 hrs at 37°C, 5% CO_2_. Cells were washed with complete RPMI medium and rested for 2 hrs in fresh medium without antibiotic-antimycotic mix before infection. For infection, 5 x 10^5^ macrophages were infected with 5 x 10^5^ mycobacteria to achieve an MOI of 1:1 in 24 well plates for 4 hrs in triplicates [31]. Following infection, the extracellular bacteria were removed by overlaying the cells with RPMI medium containing 200 μg/ml amikacin for 2 hrs. At designated time points, day 0 (4 hrs), 2, 4 and 6, macrophages were lysed by the addition of 0.025% SDS and intracellular bacteria were enumerated by plating appropriate dilutions on MB7H11 agar. Colonies were counted after 4 weeks of incubation at 37°C and the data was expressed as CFU/ml. 

### Experimental animals

 Pathogen-free outbred female guinea pigs (200-300 g) of the Duncan-Hartley strain were procured from Disease Free Small Animal House Facility, Lala Lajpat Rai University, Hissar, India. The animals were housed in individually ventilated cages and were provided with food and water *ad libitum* in a BSLIII facility at University of Delhi South Campus (UDSC), New Delhi, India. 

### Ethics statement

Guinea pig experiments included in this manuscript were reviewed and approved by the Institutional Animal Ethics Committee of University of Delhi South Campus, New Delhi, India (Ref. No. IAEC/AKT/Biochem/UDSC/24.08.2010). All animals were routinely cared for, according to the guidelines of CPCSEA (Committee for the Purpose of Control and Supervision of Experiments on Animals), India. Guinea pigs were vaccinated intradermally with mycobacterial strains by injecting not more than 100 μl and were euthanized, whenever required, by CO_2_ asphyxiation and all efforts were made to ameliorate animal suffering.

### Influence of deletion of phosphatase genes on the pathogenicity of *M.tuberculosis*

To evaluate whether the Mtb∆*mms* mutant was sufficiently attenuated for its use as a vaccine, animals (n=6) were inoculated intradermally (i.d.) with 5 x 10^5^ bacilli of either *M.tuberculosis* or Mtb∆*mms* or BCG in 100 μl of saline. Animals were euthanized at 4 weeks and 10 weeks post inoculation by CO_2_ asphyxiation. Lungs, liver and spleen were scored for gross pathological damage such as tissue involvement, areas of inflammation, extent of necrosis and number/size of tubercles due to infection. The scores given to these organs were graded from 1-4 and were based on the modified Mitchison scoring system [32]. For histopathological evaluation, the right lung and a portion of left dorsal lobe of liver were removed and fixed in 10% buffered formalin. 5 µm thick sections of formalin fixed, paraffin embedded lung tissues were stained with haemotoxylin and eosin (H & E). The tissues were coded and the coded samples were evaluated by a certified pathologist having no knowledge of the experimental groups. Left caudal lung lobe and caudal portion of spleen were aseptically removed for the measurement of the bacillary load. The specific portions of lungs and spleen were weighed and homogenized separately in 5 ml saline by using a polytron homogenizer. Appropriate dilutions of the homogenates were plated on to MB7H11 agar plates in duplicates and incubated at 37°C for 3-4 weeks. The number of colonies was counted and expressed as mean log_10_ CFU/organ.

### Evaluation of protective efficacy of MtbΔ*mms* against *M.tuberculosis* infection

Guinea pigs were divided into 3 groups (n=8) and the animals were immunized intradermally with 5 x 10^5^ CFU of either (i) BCG or (ii) Mtb*∆mms* in 100 μl of saline. In the control group, guinea pigs were injected with 100 μl of saline. Twelve weeks post immunization, guinea pigs were infected with a low dose of virulent *M.tuberculosis* via the respiratory route in an aerosol chamber (Inhalation Exposure System, Glascol Inc.), pre calibrated to deliver 10-30 bacilli in lungs per animal. Guinea pigs were euthanized at 4 weeks and 12 weeks after challenge and evaluated for bacterial load, gross pathological and histopathological changes in various organs as described in the previous section. A significant reduction in these parameters in vaccinated animals was considered as a protective effect of the vaccine.

### Statistical analyses

 For comparison between the groups, Non-parametric Kruskal–Wallis test followed by Mann-Whitney U-test, One-way analysis of variables (ANOVA) with Tukey post-test, Two-way ANOVA with Bonferroni multiple comparison test and student’s *t*-test were employed, wherever appropriate. Differences were considered significant when *p*<0.05. For statistical analyses and generation of graphs, Prism 5 software (Version 5.01; GraphPad Software Inc., CA, USA) was used.

## Results

### Functional disruption of *mptpA* and *sapM* in Mtb∆*mptpB* and characterization of the multigene mutant

**Figure 1:**
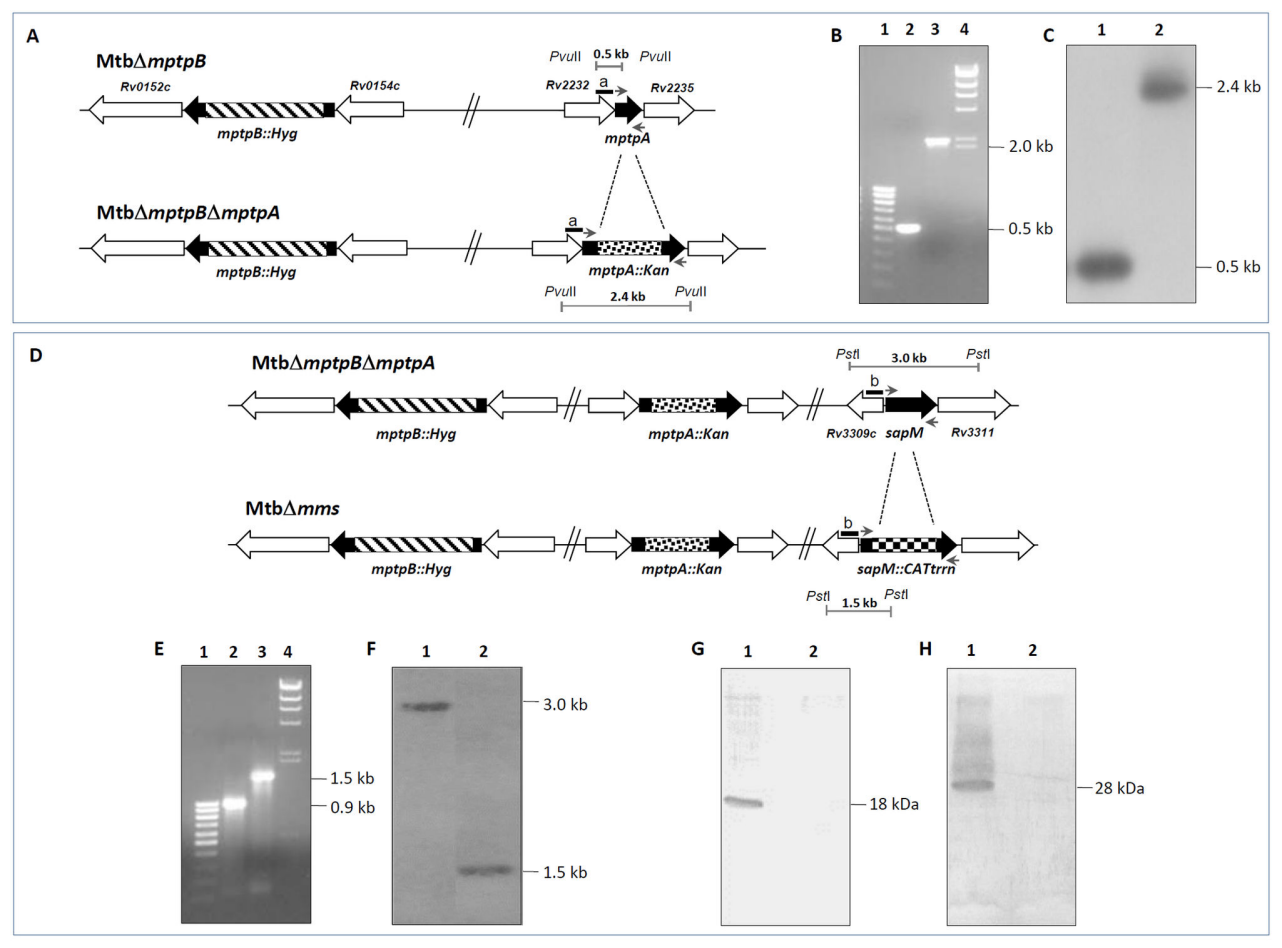
Construction and characterization of Mtb∆*mms*. (A) Disruption of *mptpA* in MtbΔ*mptpB* by homologous recombination. The figure depicts the deletion of *mptpA* by *mptpA*::*kan* AES to generate MtbΔ*mptpB*Δ*mptpA*. Solid bar (a) depicts the region of probe binding for Southern hybridization. *kan*- Kanamycin resistance cassette. (B) Confirmation of disruption of *mptpA* in Mtb∆*mptpBmptpA* was carried out by PCR by employing *mptpA* gene specific primers. A 0.5 kb amplification product was obtained with Mtb∆*mptpB* DNA as template (lane 2) and 2.0 kb amplification product was obtained with MtbΔ*mptpB*Δ*mptpA* DNA as template (lane 3). 100 bp ladder (lane 1) and λ*Hin*dIII ladder (lane 4). (C) Confirmation of *mptpA* deletion in MtbΔ*mptpB*∆*mptpA* by Southern hybridization. 200 bp at 5′ termini of *mptpA* was used as probe (a). Genomic DNA of MtbΔ*mptpB* as well as MtbΔ*mptpB*Δ*mptpA* was digested with *Pvu*II, separated on a 1.0% agarose gel, transferred to nylon membrane and probed with DIG-labeled DNA. The MtbΔ*mptpB* strain (lane 1) showed a hybridization signal at 0.5 kb as expected. The presence of 2.4 kb band in the case of MtbΔ*mptpB*Δ*mptpA* (lane 2) confirmed that allelic exchange had occurred at the *mptpA* locus. (D) Disruption of *sapM* in Mtb∆*mptpB*∆*mptpA* by homologous recombination. The figure depicts the deletion of *sapM* by *sapM*::*CATtrrn* AES to generate MtbΔ*mms*. Solid bar (b) depicts the region of probe binding for Southern hybridization. *CATtrrn*-Choloramphenicol resistance cassette. (E) Confirmation of disruption of *sapM* in Mtb∆*mms* was carried out by PCR by employing *sapM* gene specific primers. A 0.9 kb amplification product was obtained with Mtb∆*mptpB*∆*mptpA* DNA as template (lane 2) and 1.5 kb amplification product was obtained with MtbΔ*mms* mutant DNA as template (lane 3). 100 bp ladder (lane 1) and λ*Hin*dIII ladder (lane 4). (F) Confirmation of *sapM* deletion in MtbΔ*mms* by Southern hybridization. 200 bp at 5′ termini of *sapM* was used as probe (b). Genomic DNA of MtbΔ*mptpB*Δ*mptpA* as well as MtbΔ*mms* strain was digested with *Pst*I, separated on a 1.0% agarose gel, transferred to nylon membrane and probed with DIG-labeled DNA. The MtbΔ*mptpB*Δ*mptpA* strain (lane 1) showed a hybridization signal at 3.0 kb as expected. The presence of 1.5 kb band in the case of MtbΔ*mms* strain (lane 2) confirmed that allelic exchange had occurred at the *sapM* locus. (G) Confirmation of *mptpA* deletion in Mtb∆*mms* by immunoblot analysis. 50 μg of culture filtrate proteins of Mtb∆*mptpB* (lane 1) and MtbΔ*mms* (lane 2) were loaded onto a 12.5% polyacrylamide gel and subjected to electrophoresis. MptpA was detected by immunoblot analysis by using anti-MptpA polyclonal antisera. MptpA protein migrated as an 18 kDa protein band (lane 1). Disruption of *mptpA* in MtbΔ*mms* was confirmed by the absence of this 18 kDa band (lane 2). (H) Confirmation of *sapM* deletion in Mtb∆*mms* by immunoblot analysis. 50 μg of culture filtrate proteins of Mtb∆*mptpB* (lane 1) and MtbΔ*mms* (lane 2) were loaded onto a 12.5% polyacrylamide gel and subjected to electrophoresis. SapM was detected by immunoblot analysis by using anti-SapM polyclonal antisera. SapM protein migrated as a 28 kDa protein band (lane 1). Disruption of *sapM* in MtbΔ*mms* was confirmed by the absence of this 28 kDa band (lane 2).

To generate triple gene mutant of *M.tuberculosis*, we first disrupted *mptpA* in Mtb∆*mptpB* (published from our laboratory previously, [26]) to generate Mtb∆*mptpB*∆*mptpA* (Figure 1A). Deletion of *mptpA* was confirmed by three approaches (1). PCR by using *mptpA* gene specific primers (Table 1, Figure 1B). In the case of Mtb∆*mptpB*, a 0.5 kb amplicon representing the complete *mptpA* gene was amplified as expected, while in the case of Mtb∆*mptpB*∆*mptpA*, an amplicon of 2.0 kb was observed indicating the disruption of *mptpA* by Kanamycin resistance cassette (2). Southern hybridization. In the case of MtbΔ*mptpB* strain, the probe hybridized to a 0.5 kb (lane 1) *Pvu*II fragment whereas disruption of *mptpA* gene by Kanamycin resistance cassette resulted in a signal at 2.4 kb (lane 2) in the MtbΔ*mptpB*Δ*mptpA* strain (Figure 1C) (3). Nucleotide sequencing. Both 0.5 kb and 2.0 kb amplification products were DNA sequenced that further confirmed the disruption of *mptpA* in MtbΔ*mptpB*Δ*mptpA*. Further, the *sapM* gene was deleted in MtbΔ*mptpB*Δ*mptpA* by employing linear AES to generate MtbΔ*mms* (Figure 1D). The triple gene mutant was confirmed by PCR by employing *sapM* gene specific primers (Table 1, Figure 1E). The primers yielded an amplicon of 0.9 kb in Mtb∆*mptpB*∆*mptpA*, however, the deletion of *sapM* gene resulted in a PCR amplicon of 1.5 kb in Mtb∆*mms* (Figure 1E) (2). Southern hybridization. The probe in the MtbΔ*mptpB*Δ*mptpA* hybridized to a 3.0 kb (lane 1) *Pvu*II fragment whereas disruption of *sapM* gene by Chloramphenicol resistance cassette resulted in a signal at 1.5 kb (lane 2) in the MtbΔ*mms* strain (Figure 1F) (3). Nucleotide sequencing. Both 0.9 kb and 1.5 kb amplification products were DNA sequenced that further confirmed the disruption of *sapM* in MtbΔ*mms*. Deletion of *mptpA* and *sapM* was further confirmed by immunoblot analysis by using polyclonal antibodies raised against MptpA and SapM. As shown in Figure 1G and Figure 1H, we did not observe any expression of MptpA and SapM in MtbΔ*mms*.

### Disruption of Phosphatases Does Not Alter the Lipid Profile of MtbΔ*mms*

To ascertain whether the disruption of phosphatase genes had any influence on the lipid composition of the mutant, we performed a total lipid analysis of the parental as well as the mutant strain by TLC. *M.tuberculosis* and MtbΔ*mms* were analysed for the well known characterstic lipids of the tubercle bacillus. The apolar and polar lipid fractions were extracted and assayed for phthiocerol dimycocerosate (PDIM), triacylglycerol (TAG), mycolic acids, free fatty acids, diacylglycerol (DAG), diacyltrehalose (DAT), trehalose monomycolate (TMM), trehalose dimycolate (TDM), glucose monomycolate (GMM), sulfolipids (SL), phosphatidylinositol (PI) and phosphatidylinositol mannoside (PIMs) and phospholipids (P) by TLC. Equivalent amounts of apolar as well as polar lipids from both *M.tuberculosis* and MtbΔ*mms* were spotted on TLC plates and analysed for different lipid fractions (Table S1). 

**Figure 2:**
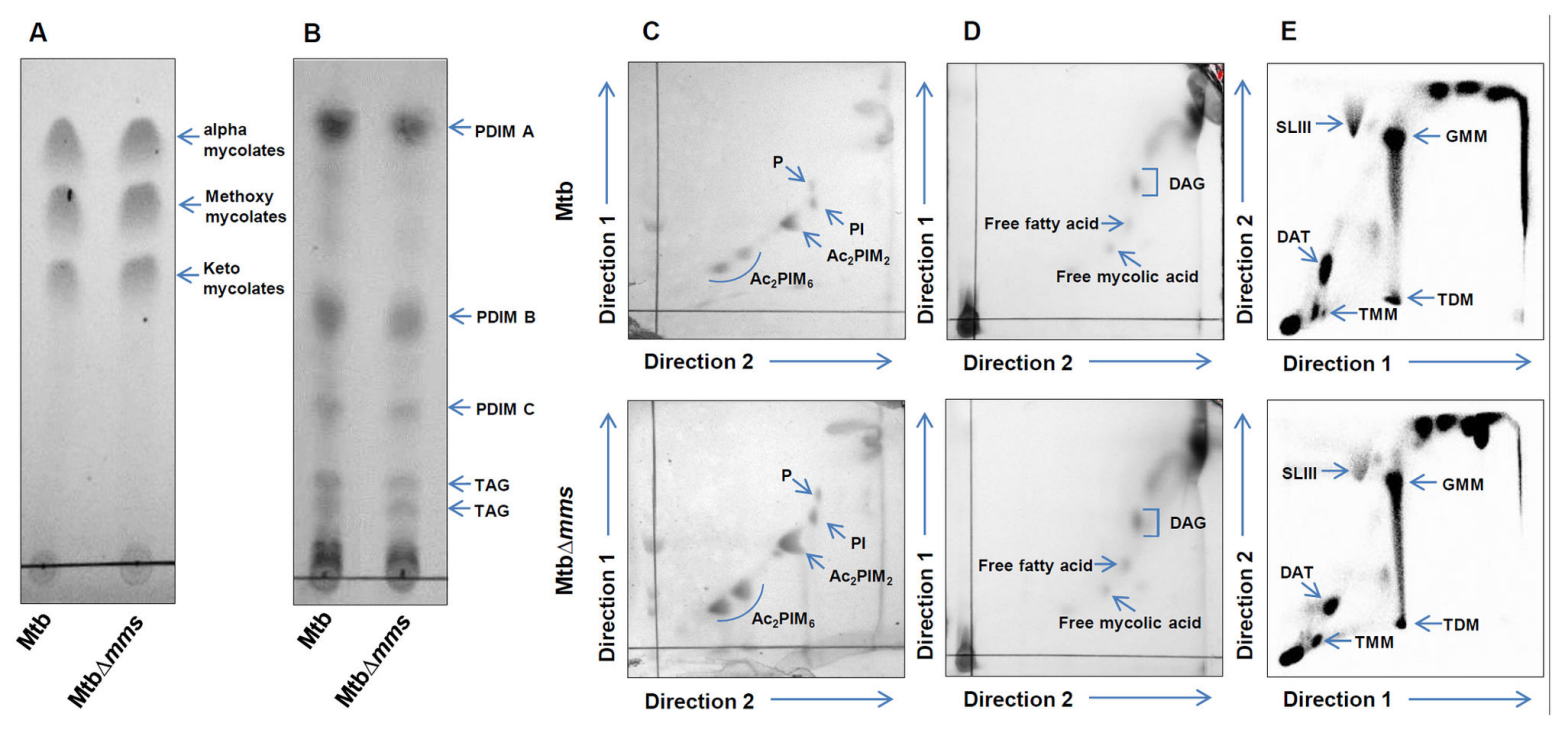
Lipid analysis of *M.tuberculosis* and MtbΔ*mms*. (A) Total mycolic acids extracts from *M.tuberculosis* (Mtb) and MtbΔ*mms* separated by TLC in hexane: ethyacetate (95: 5 v/v) seven times and detected with 20% sulfuric acid in ethanol followed by charring at 100°C. Single dimension TLC depicting alpha-, methoxy- and keto- types of mycolic acids. (B) Apolar lipid extracts from Mtb and MtbΔ*mms* separated by TLC in petroleum ether: diethyl ether (90: 10 v/v) and detected with 10% phosphomolybdic acid in ethanol followed by charring at 100°C showing PDIMs and TAG. (C) Lipid extracts from Mtb and MtbΔ*mms* separated by two dimensional TLC in chloroform: methanol: water (60: 30: 6 v/v/v) in 1^st^ direction and chloroform: acetic acid: methanol: water (40: 25: 3: 6 v/v/v/v) in 2^nd^ direction. PIMs, PI and P were detected with 2% orcinol in 10% sulphuric acid followed by charring. (D) Lipid extracts from Mtb and MtbΔ*mms* separated by two dimensional TLC in chloroform: methanol (96: 4 v/v) in 1^st^ direction and toluene: acetone (80: 20 v/v) in 2^nd^ direction. DAG, free fatty acids and free mycolic acids were detected with 10% phosphomolybdic acid in ethanol followed by charring. (E) TLC analysis of lipid extracts from [14C] acetate-labeled bacterial cell of Mtb and MtbΔ*mms*. Lipid extracts were dissolved in solvent and run in chloroform: methanol: water (100: 14: 0.8 v/v/v) in 1^st^ direction and chloroform: acetone: methanol: water (50: 60: 2.3: 3 v/v/v/v) in 2^nd^ direction. GMM, SLIII, DAT, TMM and TDM were visualized with a Typhoon FLA 700 Phosphorimager. PDIM- phthiocerol dimycocerosate, TAG- triacylglycerol, DAG- diacylglycerol, GMM- glucose monomycolate, SL- sulfolipids, DAT- diacyltrehalose, TMM- trehalose monomycolate, TDM- trehalose dimycolate, PIM- phosphatidylinositol mannoside, PI- phosphatidylinositol, P- phospholipids. TLC analysis of the lipids of *M.tuberculosis* and MtbΔ*mms* exhibited a similar and usual lipid profile with respect to the mycobacterial lipid components.

 TLC analysis of the lipids of *M.tuberculosis* and MtbΔ*mms* exhibited a similar and usual lipid profile with respect to the mycobacterial lipid components. *M.tuberculosis* produces three classes of mycolic acids: alpha-, keto- and methoxy- mycolic acids [33]. Analysis of total mycolic acids extracted from both *M.tuberculosis* and MtbΔ*mms* by single dimension TLC exhibited that there was no significant difference in total or alternate types of mycolic acids (Figure 2A). In addition, we observed similar accumulation of structural variants of DIM and TAGs as described by Giovannini et al [34] (Figure 2B). Two dimensional TLC indicated the equivalent presence of both apolar (DAG, TMM, TDM, SL, GMM, DAT) and polar lipids (PIMs, PI and P) in both *M.tuberculosis* and MtbΔmms as described previously by Bhatt et al [35] (Figure 2C, Figure 2D and Figure 2E). Hence, our observation demonstrated that the lipid profile of *M.tuberculosis* and MtbΔ*mms* was similar with no notable differences.

### Mtb∆*mms* exhibits a severe growth defect in human THP-1 macrophages

**Figure 3:**
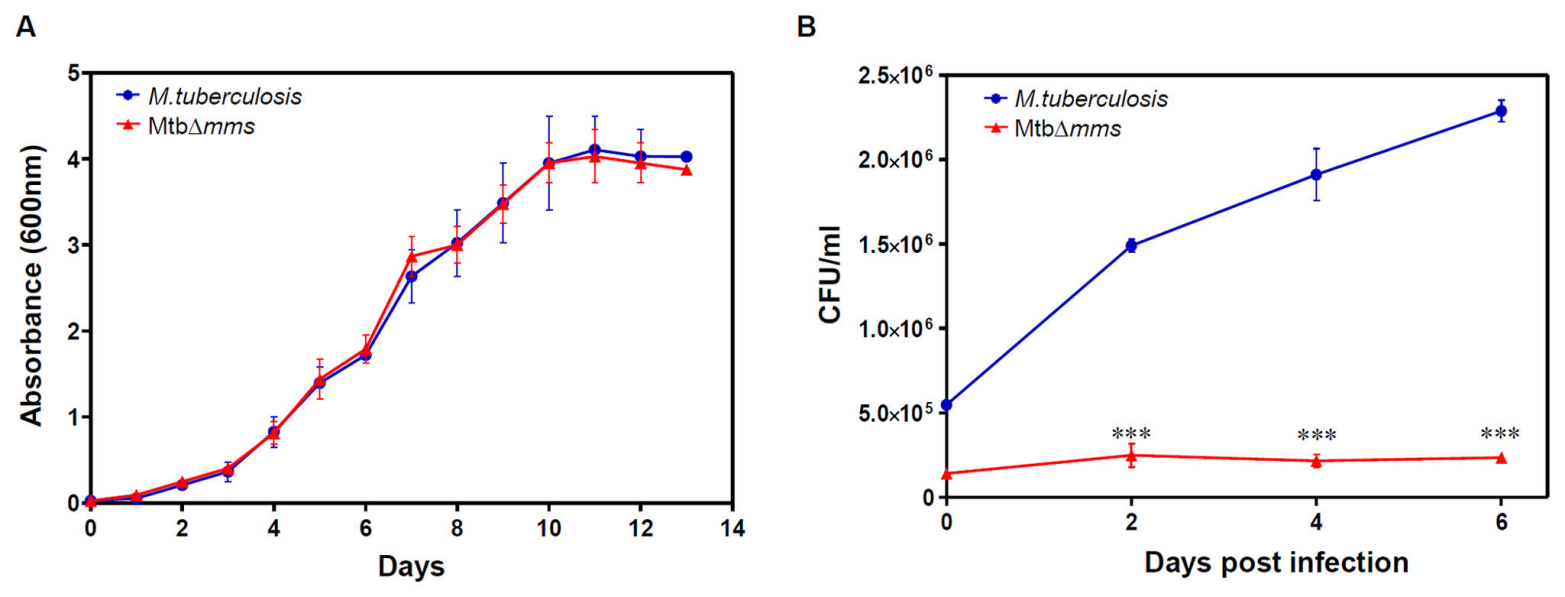
Influence of deletion of phosphatase genes on the growth and survival of *M.tuberculosis* *in* *vitro*. (A) Growth of *M.tuberculosis* and MtbΔ*mms* in broth culture. *M.tuberculosis* and MtbΔ*mms* strains were cultured separately in MB7H9 medium at 37°C/200 rpm for 13 days. *M.tuberculosis* and MtbΔ*mms* exhibited similar growth pattern in MB7H9 broth culture. (B) Growth of *M.tuberculosis* and MtbΔ*mms* in human THP-1 macrophages. THP-1 cells were infected with *M.tuberculosis* or Mtb∆*mms* mutant separately at an MOI of 1:1. The number of intracellular viable bacteria was determined for 6 days. The figure represents the CFU data of *M.tuberculosis* and MtbΔ*mms* obtained on MB7H11 plates. Mtb∆*mms* mutant exhibited a significant attenuation in its growth when compared with *M.tuberculosis*, till 6 days post infection. The experiment was repeated thrice with three independent samples each time. ∗∗∗p<0.001 (Two-way ANOVA). The values are represented as the means (±SE).

 Next, we compared the growth characteristics of MtbΔ*mms* and the parental strain in MB7H9 medium and in THP-1 cells. As shown in Figure 3A, we did not observe any difference in the growth characteristics of MtbΔ*mms* and the parental strain in MB7H9, however, a significant difference was observed in the growth kinetics between these two strains in THP-1 macrophages. We observed that Mtb∆*mms* displayed a significantly reduced ability (~2.89 fold difference) to infect macrophages in comparison to the parental strain. Moreover, while *M.tuberculosis* continued to grow normally for 6 days, Mtb∆*mms* exhibited no sign of growth during this time period demonstrating that the deletion of 3 phosphatases rendered the mutant completely incapable of growing in the macrophages (∗∗∗p<0.001) (Figure 3B). These results demonstrate the importance of *mptpA*, *mptpB* and *sapM* in the growth and survival of *M.tuberculosis* in the human macrophages.

### Deletion of phosphatase genes leads to the attenuation of *M.tuberculosis*

**Figure 4:**
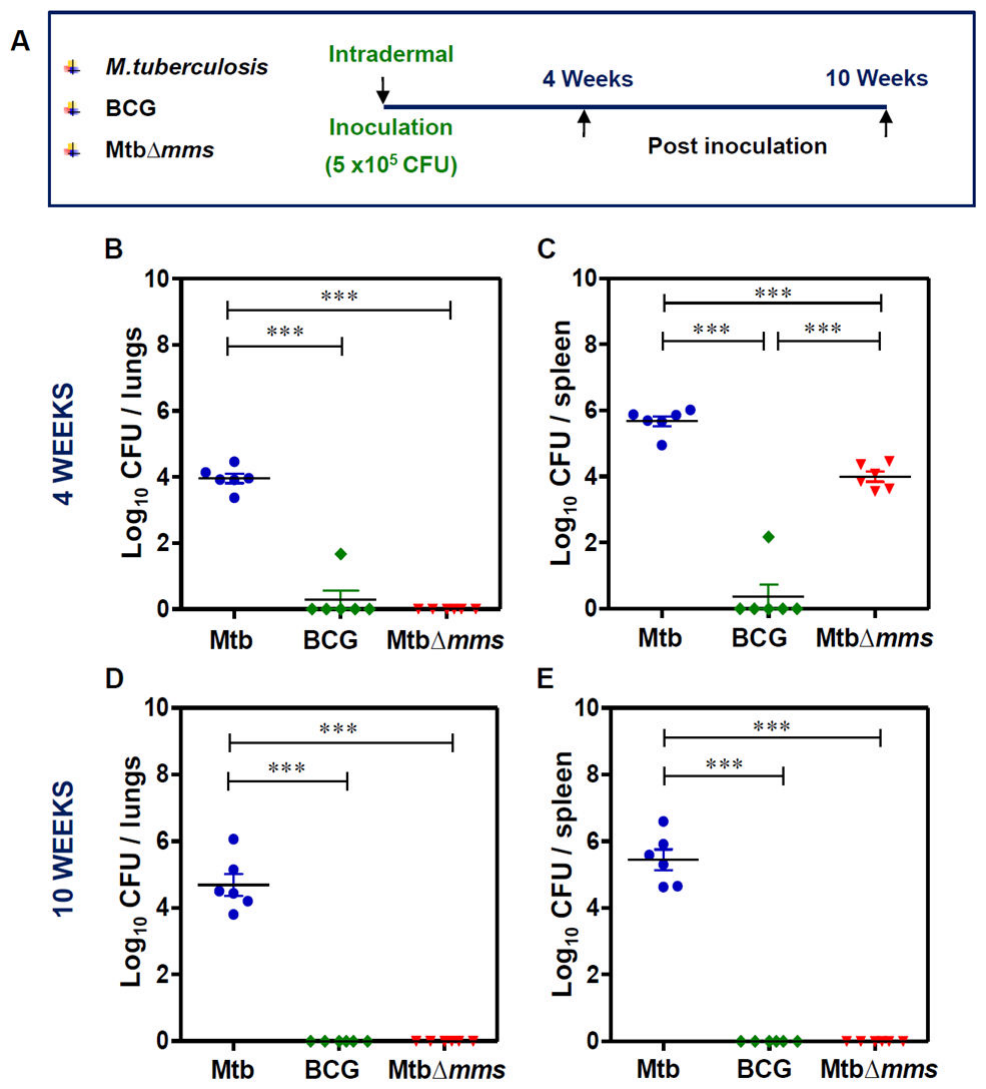
Bacillary load in the organs of guinea pigs post inoculation with various strains. (A) Experimental protocol for evaluating the attenuation of MtbΔ*mms*. (B, C) Bacillary load in the lungs and spleens of guinea pigs inoculated with *M.tuberculosis* (Mtb), BCG or Mtb∆*mms* at 4 weeks post inoculation. (D, E) Bacillary load in the lungs and spleens of guinea pigs inoculated with Mtb, BCG or Mtb∆*mms* at 10 weeks post inoculation. At 10 weeks post inoculation, no bacterial colony was obtained from the lung and spleen homogenates of BCG or Mtb∆*mms* inoculated animals on MB7H11 agar. Each data point represents the Log_10_ CFU value for an individual animal (n=6) and the bar depicts mean (±SE) for each group. ∗∗∗p<0.001 (One way ANOVA).

 To evaluate whether the deletion of three phosphatases had rendered the Mtb∆*mms* mutant sufficiently attenuated for its use as a vaccine, animals were inoculated with either *M.tuberculosis* or BCG or Mtb∆*mms* strain (Figure 4A). At 4 weeks post inoculation, we observed the maximum bacillary load of 3.96 log_10_ CFU in the lungs of *M.tuberculosis* infected animals, as compared to negligible bacillary load of 0.28 log_10_ CFU in the lungs of BCG treated animals. No bacilli were detectable in the lungs of Mtb∆*mms* inoculated animals (Figure 4B). However, when the splenic bacillary counts were analyzed, we observed 5.68 log_10_ CFU, 0.36 log_10_ CFU and 3.99 log_10_ CFU in the animals inoculated with *M.tuberculosis*, BCG and Mtb∆*mms*, respectively (Figure 4C). Hence, during this initial phase, Mtb∆*mms* showed some growth in the spleens of animals, although it was ~70 fold less as compared to the parental strain. At 10 weeks post inoculation, a bacillary load of 4.69 log_10_ CFU was observed in the lungs of *M.tuberculosis* infected animals. However, at this time point, no bacilli were recovered from the lungs of the animals inoculated with either Mtb∆*mms* or BCG (Figure 4D). In spleens, we observed a bacillary count of 5.45 log_10_ CFU in *M.tuberculosis* infected animals. However, no bacilli were recovered from the spleens of animals inoculated with either Mtb∆*mms* or BCG (Figure 4E). 

 Although, Mtb∆*mms* exhibited some growth in the spleens of the inoculated animals, the bacilli were recovered only during the initial phase (4 weeks post inoculation) and the bacillary load was only 1.4% of that observed in the case of *M.tuberculosis* infected animals (70 fold fewer bacilli in Mtb∆*mms* inoculated animals). Further, on extending the time post inoculation, no Mtb∆*mms* bacilli were recovered in spleens as well as in lungs. Thus, based on these observations, it appeared that as a result of deletion of the phosphatase genes, Mtb∆*mms* was sufficiently attenuated for growth in the host tissues and could be safely used as a vaccine candidate.

### Deletion of phosphatase genes renders *M.tuberculosis* incapable of causing pathology in guinea pigs at 10 weeks post inoculation

**Figure 5:**
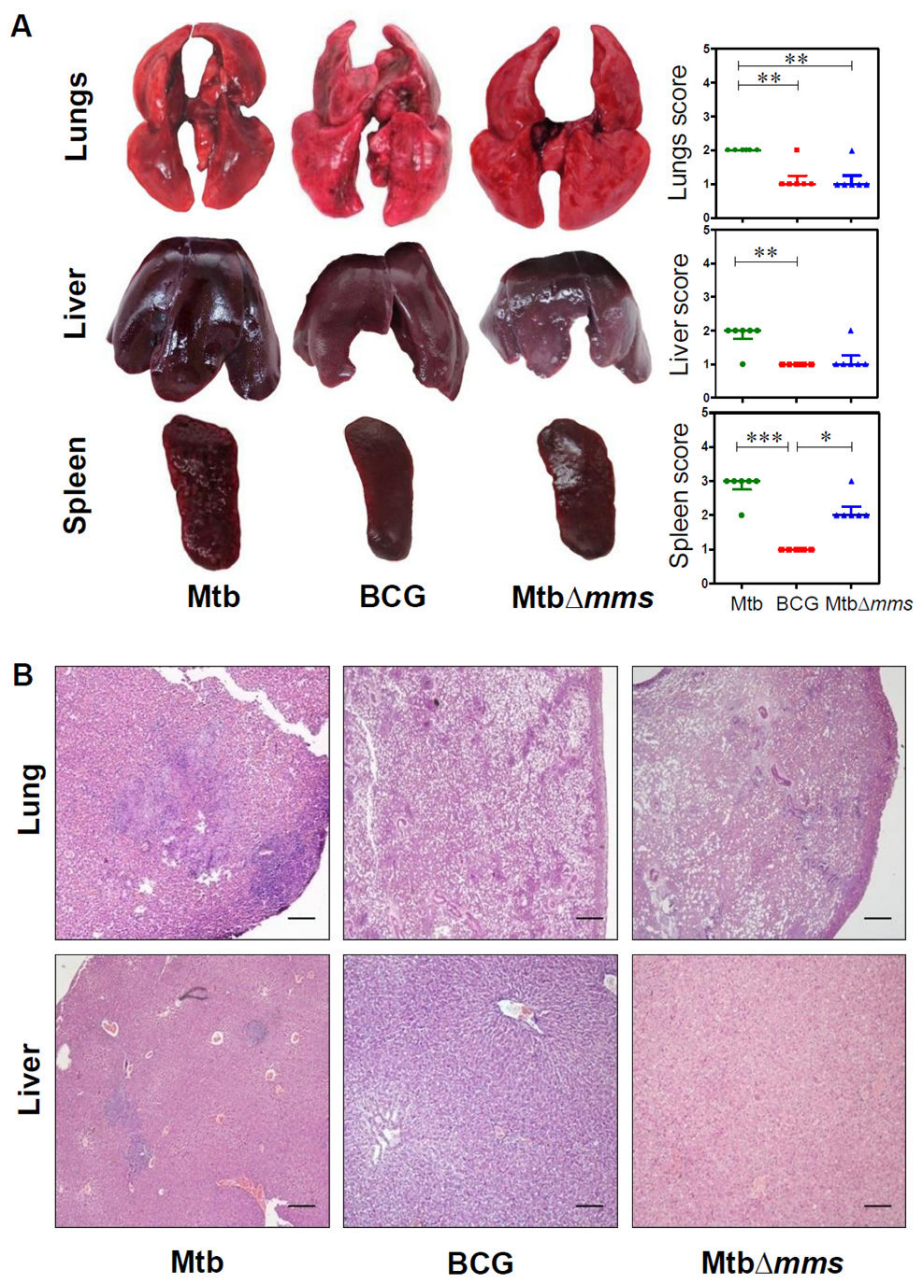
Gross pathology and histopathology of guinea pig organs inoculated with various strains at 4 weeks. (A) Representative photographs of lungs, liver and spleen of individual animal (n=6) euthanized at 4 weeks post inoculation with either *M.tuberculosis* (Mtb), BCG or MtbΔ*mms*. The graphical representation of gross scores of various organs is also shown alongside. The bar depicts median for each group. ∗p<0.05; ∗∗p<0.01 and ∗∗∗p<0.001 (Kruskal Wallis test). (B) The representative photomicrographs of the lung and liver tissues stained with Haematoxylin-Eosin staining (H&E). The figure depicts 20x magnification of representative photomicrographs of the organs of animals inoculated with either Mtb, BCG or MtbΔ*mms* euthanized at 4 weeks post inoculation. The scale bars depict 500 μm for the lung as well as liver sections. MtbΔ*mms* inoculated animals displayed negligible lung and hepatic pathology. However, in the case of spleens, Mtb∆*mms* inoculation resulted in some pathological damage, although, the damage was considerably less in comparison to the *M.tuberculosis* infected animals.

**Figure 6:**
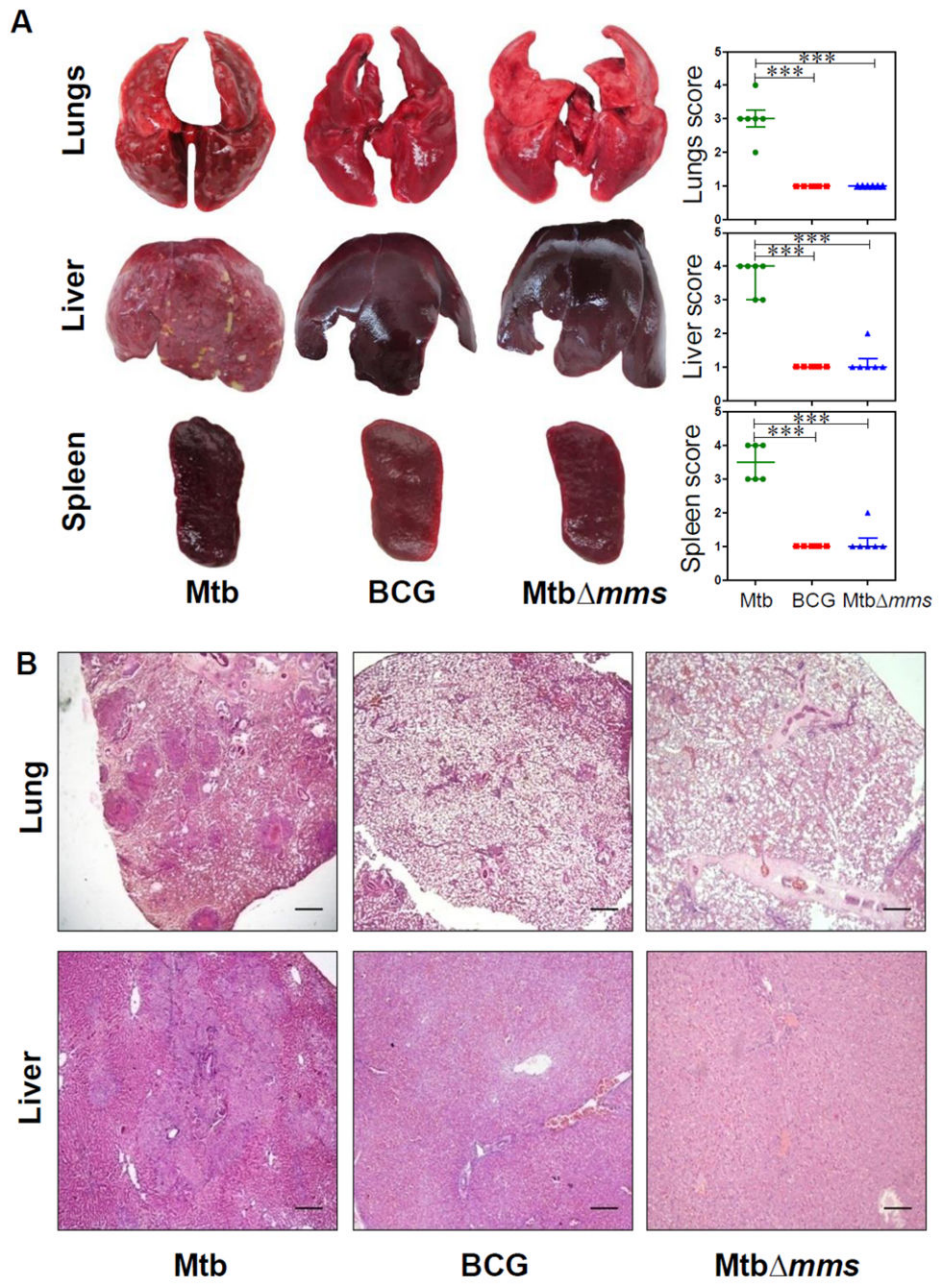
Gross pathology and histopathology of guinea pig organs inoculated with various strains at 10 weeks. (A) Representative photographs of lungs, liver and spleen of individual animal (n=6) euthanized at 10 weeks post inoculation with either *M.tuberculosis* (Mtb), BCG or MtbΔ*mms*. The graphical representation of gross scores of various organs is also shown alongside. The bar depicts median for each group. ∗∗∗p<0.001 (Kruskal Wallis test). (B) The representative photomicrographs of the lung and liver tissues stained with Haematoxylin-Eosin staining (H&E). The figure depicts 20x magnification of representative photomicrographs of the organs of animals inoculated with either Mtb, BCG or MtbΔ*mms* euthanized at 10 weeks post inoculation. The scale bars depict 500 μm for the lung as well as liver sections. MtbΔ*mms* inoculated animals displayed complete restoration of pulmonary and hepatic parenchyma with negligible pathological damage to the tissues at 10 weeks post inoculation.

 The gross pathological changes observed in the organs of the animals at 4 weeks and 10 weeks post inoculation with Mtb∆*mms* were commensurate with the bacillary load observed. At 4 weeks post inoculation, the extent of damage observed in the case of *M.tuberculosis* inoculated animals was found to be maximum amongst all the groups with numerous small sized tubercles along with scattered areas of necrosis in all the organs (score: 2 in lungs and liver and 3 in spleen), indicating progressive pulmonary and extra-pulmonary disease (Figure 5A). However, in the case of BCG inoculation, no pathology was observed in the lungs, liver or spleen as expected (score: 1 in all the organs). In the case of inoculation with Mtb∆*mms*, most of the animals displayed negligible lung and hepatic pathology (score: 1) with predominantly scanty and extremely small necrotic lesions. However, in the case of spleen, Mtb∆*mms* inoculated animals were allotted intermediate score (score: 2) in comparison to other two groups. This indicated that Mtb∆*mms* inoculation resulted in some pathological damage to spleens, although, the damage was considerably less in comparison to the *M.tuberculosis* infected animals (Figure 5A). When the animals were evaluated at 10 weeks post inoculation, in the case of *M.tuberculosis* infected animals, as expected, the extent of damage was more than that observed at 4 weeks (score: 3 in lungs, 4 in liver and 3-4 in spleen) with extensive involvement and numerous large sized tubercles effacing the entire organs (Figure 6A). However, the animals inoculated with either BCG or Mtb∆*mms* displayed normal lungs, liver and spleen phenotype (score: 1) with no pathological damage (Figure 6A).

 To evaluate the histopathological changes in the lungs and liver of guinea pigs inoculated with *M.tuberculosis*, BCG or Mtb∆*mms*, the tissue sections were stained with haematoxylin and eosin. At 4 weeks post inoculation, the lungs of *M.tuberculosis* infected animals exhibited granulomatous infiltration with caseating necrotic granulomas effacing the pulmonary parenchyma (Figure 5B). Inoculation with either BCG or Mtb∆*mms* resulted in a negligible granulomatous infiltration in lungs when compared with *M.tuberculosis* infected animals. Liver of *M.tuberculosis* infected animals displayed moderate involvement of tissues with few granulomatous lesions. However, BCG or Mtb∆*mms* inoculated animals exhibited a normal hepatic tissue organization with clearly visible portal triad (Figure 5B). At 10 weeks post inoculation, the lungs of *M.tuberculosis* infected animals exhibited advanced stage granuloma with extensive coalescence of multiple granulomas. The lung granulomas were characterized by extensive necrosis resulting in the loss of lung micro-architecture (Figure 6B). However, in the case of inoculation with either BCG or Mtb∆*mms*, the alveolar and bronchiolar structures of the lungs were well preserved with no sign of active disease. Liver of *M.tuberculosis* infected animals exhibited extensive pathological damage with multiple coalescing granulomas effacing the entire hepatic tissues, where as BCG or Mtb∆*mms* inoculated animals exhibited normal hepatic tissue organization with clearly visible hepatic lobules (Figure 6B). 

### Mtb∆*mms* vaccination limits *M.tuberculosis* multiplication in the lungs of guinea pigs

**Figure 7:**
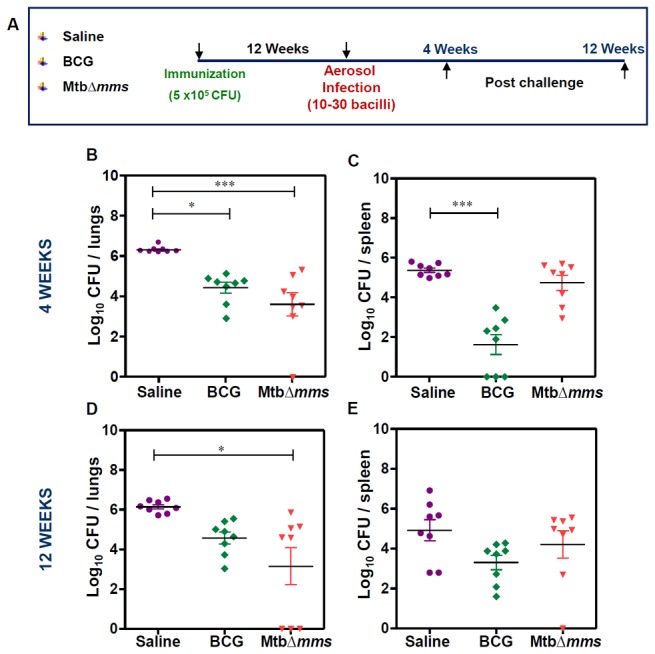
Determination of bacillary load in guinea pig organs after immunization with various strains. (A) Experimental protocol for evaluating the protective efficacy of MtbΔ*mms* against *M.tuberculosis* infection. (B, C) The bacillary load in the lungs and spleens of guinea pigs (n=8) vaccinated with BCG, Mtb∆*mms* or saline at 4 weeks after *M.tuberculosis* challenge. (D, E) The bacillary load in the lungs and spleens of guinea pigs (n=8) vaccinated with BCG, Mtb∆*mms* or saline at 12 weeks after challenge. Mtb∆*mms* imparted a significant protection as compared to the sham immunized animals in the lungs of vaccinated animals. Each data point represents the Log_10_ CFU value for an individual animal and the bar depicts mean (±SE) for each group. ∗p<0.05 and ∗∗∗p<0.001 (One-way ANOVA).

 As the Mtb∆*mms* mutant appeared to be safe for its use as a vaccine candidate, we next evaluated its protective efficacy against *M.tuberculosis* challenge. For this, guinea pigs were vaccinated with either BCG or Mtb∆*mms* and were infected with 10-30 *M.tuberculosis* bacilli by aerosol route at 12 weeks post vaccination. As a control, one group of guinea pigs was sham immunized. Following 4 and 12 weeks after challenge, animals were euthanized and bacillary load in the lungs and spleens was determined (Figure 7A). At 4 weeks after challenge, the sham immunized animals exhibited 6.30 log_10_ CFU in lungs. The BCG vaccinated animals exhibited a significantly reduced CFU in lungs (4.43 log_10_ CFU) indicating 1.87 log_10_ CFU reduction (∗p<0.05) as compared to the sham-immunized animals (Figure 7B). Mtb*∆mms* vaccinated animals exhibited a bacillary load of only 3.60 log_10_ CFU in lungs indicating that the mutant also significantly reduced the pulmonary load by 2.70 log_10_ CFU (∗∗∗p<0.001) in comparison to the sham immunized animals (Figure 7B). Further, the sham immunized animals exhibited a splenic bacillary load of 5.37 log_10_ CFU (Figure 7C), while, the BCG vaccinated animals exhibited a splenic bacillary load of only 1.62 log_10_ CFU. This significant reduction in splenic bacillary load by 3.75 log_10_ CFU (∗∗∗p<0.001) demonstrated a tight control of hematogenous spread of bacilli by BCG. The splenic bacillary load in the case of Mtb∆*mms* vaccinated animals (4.73 log_10_ CFU) was 0.64 log_10_ CFU less when compared with the sham immunized animals but the difference was not significant (Figure 7C).

 On extending the time period between challenge and euthanasia to 12 weeks, the sham immunized animals exhibited a bacillary load of 6.15 log_10_ CFU in lungs (Figure 7D). Immunization with BCG resulted in 4.57 log_10_ CFU in lungs as compared to the sham immunized animals, however, the difference in the pulmonary bacillary load between BCG and sham immunized animals was statistically not significant and with the extension of time, the ability of BCG to impede bacillary multiplication met with a considerable decline. In contrast, Mtb*∆mms* vaccinated animals exhibited only 3.16 log_10_ CFU in lungs, thus indicating a significantly reduced bacillary load by 2.99 log_10_ CFU (∗p<0.05) in comparison to the sham immunized animals. These observations demonstrated that Mtb∆*mms* exhibited a more sustainable and superior protection as compared to BCG. The splenic bacillary load in the case of sham immunized animals was 4.92 log_10_ CFU (Figure 7E). Although, the splenic bacillary loads in the cases of BCG vaccination and Mtb∆*mms* vaccination were 3.30 log_10_ CFU and 4.21 log_10_ CFU, respectively, these were not significantly different in comparison to the splenic bacillary load observed in the sham immunized animals. Thus, our observations indicated that Mtb∆*mms* was not able to exhibit a significant control on the hematogenous spread at either 4 weeks or 12 weeks after challenge. 

### Mtb∆*mms* vaccination imparts protection from pathological damage in lungs

**Figure 8:**
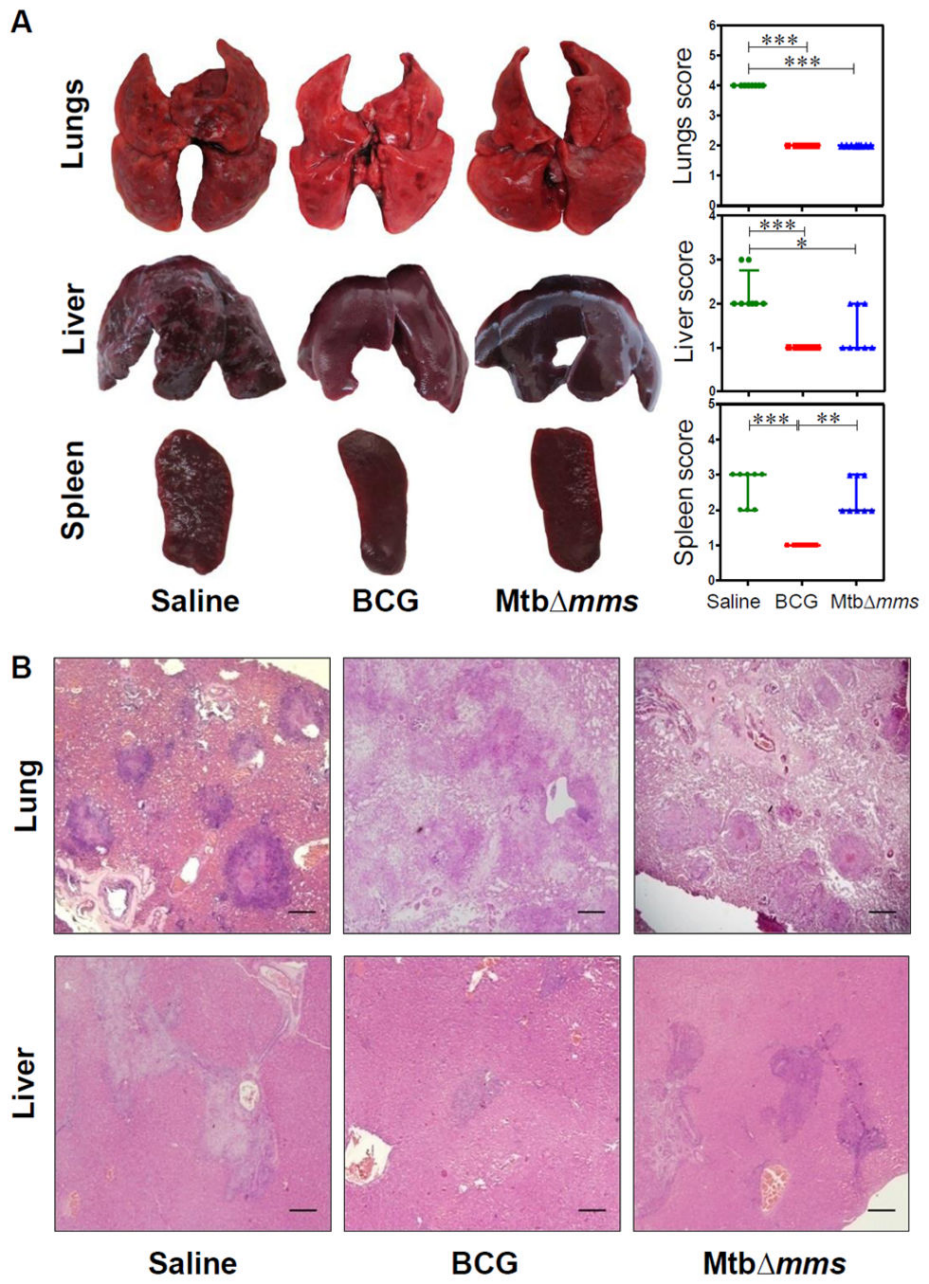
Gross pathology and histopathology of vaccinated guinea pig organs at 4 weeks after challenge. (A) Representative photographs of lungs, liver and spleen of individual animal (n=8) vaccinated with either BCG, MtbΔ*mms* or saline and euthanized at 4 weeks after challenge. The graphical representation of gross scores of various organs is also shown alongside. The bar depicts median for each group. ∗p<0.05; ∗∗p<0.01 and ∗∗∗p<0.001 (Kruskal Wallis test). (B) The representative photomicrographs of the lung and liver tissues stained with Haematoxylin-Eosin staining (H&E). The figure depicts 20x magnification of representative photomicrographs of organs of the animals vaccinated with either BCG, MtbΔ*mms* or saline and euthanized at 4 weeks after challenge. The scale bars depict 500 μm for lung as well as liver sections. Unvaccinated animals show severe pathology in lungs characterized by the presence of numerous large and small sized tubercles. However, hepatic and splenic tissues show moderate involvement. In the case of immunization with Mtb*∆mms*, the animals show moderately inflamed lungs with minimal hepatic tissue destruction similar to that observed in the case of BCG vaccination. However, spleens of Mtb∆*mms* vaccinated animals exhibit pathological damage similar to sham immunized animals.

**Figure 9:**
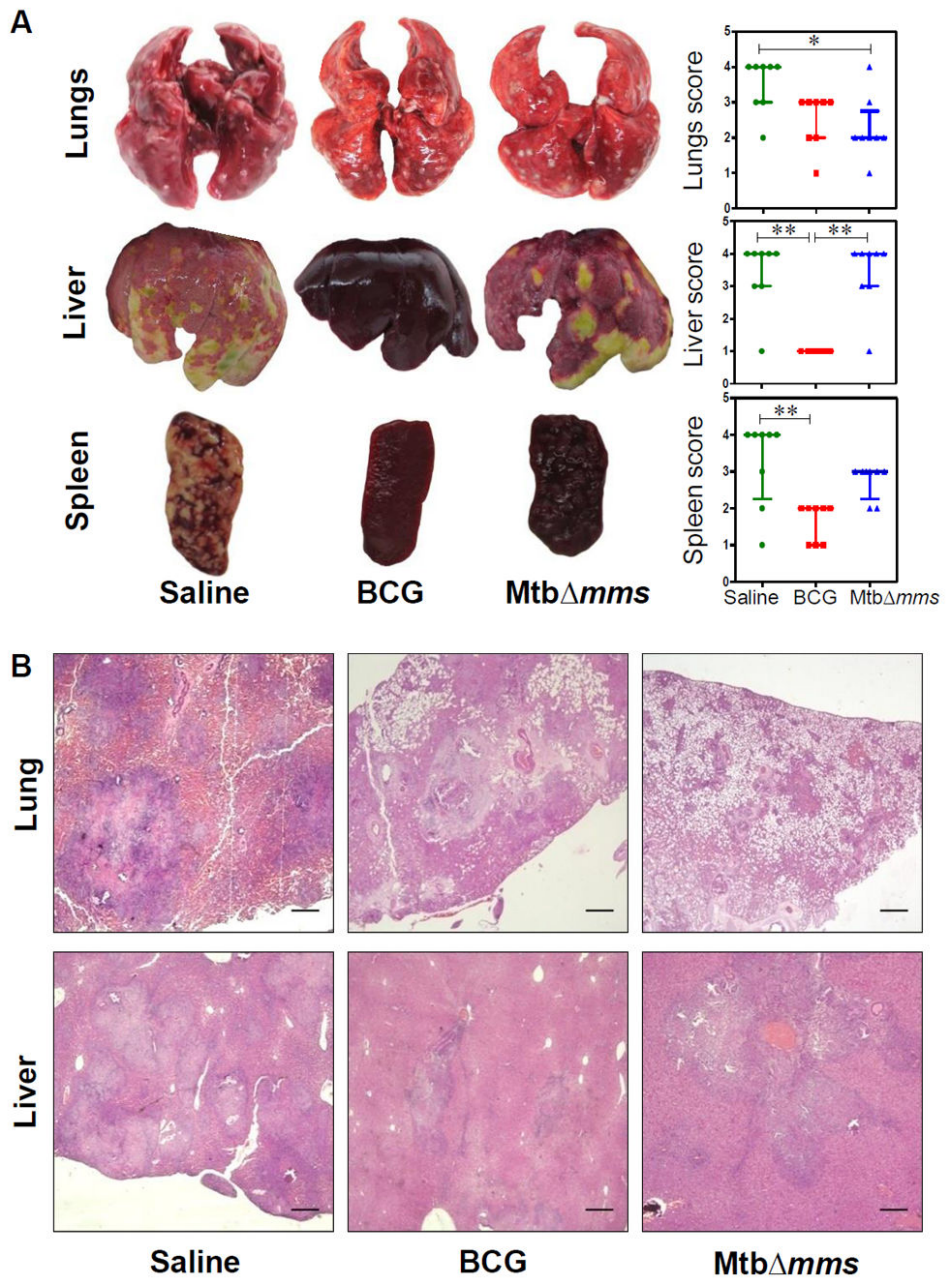
Gross pathology and histopathology of vaccinated guinea pig organs at 12 weeksafter challenge. (A) Representative photographs of lungs, liver and spleen of individual animal (n=8) vaccinated with either BCG, MtbΔ*mms* or saline and euthanized at 12 weeks after challenge. The graphical representation of gross scores of various organs is also shown alongside. The bar depicts median for each group. ∗p<0.05 and ∗∗p<0.01 (Kruskal Wallis test). (B) The representative photomicrographs of the lung and liver tissues stained with Haematoxylin-Eosin staining (H&E). The figure depicts 20x magnification of representative photomicrographs of organs of the animals vaccinated with either BCG, MtbΔ*mms* or saline and euthanized at 12 weeks after challenge. The scale bars depict 500 μm for lung as well as liver sections. Unvaccinated animals show characteristic presence of multiple coalescing granulomas with necrosis in both lungs and liver. In addition, the animals show a marked discoloration of spleen with numerous large and small sized tubercles. Mtb∆*mms* immunized animals, on the other hand show reduced granulomatous infiltration with only a few small and discrete granulomas in lungs. However, liver and spleen of Mtb∆*mms* vaccinated animals exhibit pathological damage similar to sham immunized animals.

 At 4 weeks after challenge, the sham immunized animals exhibited severe pathology in lungs characterized by the presence of numerous large and small sized tubercles (score: 4 in lungs). However, hepatic and splenic tissues exhibited moderate involvement (score: 2 in liver and 2-3 in spleen) (Figure 8A). In contrast, BCG vaccinated animals displayed significantly reduced gross lesions in the organs when compared with the unvaccinated animals (score: 2 in lungs and 1 in liver and spleen). In the case of immunization with Mtb*∆mms*, the animals exhibited moderately inflamed lungs and spleen (score: 2 in both the organs) with minimal hepatic tissue destruction (score: 1) (Figure 8A). On extending the period between challenge and euthanasia to 12 weeks, we observed an overall increase in the gross pathological damage to the organs of sham immunized animals as characterized by extensive involvement of tissue with numerous large tubercles and scattered areas of necrosis in both lungs and liver (score: 3-4). In addition, a marked discoloration of spleen with numerous large and small sized tubercles and occasional attrition of capsular structure was also observed in most of the sham immunized animals (score: 4) (Figure 9A). BCG immunized guinea pigs exhibited moderate involvement of lung and splenic tissues with small sized tubercles effacing the entire tissues (score: 2-3 in lungs and 1-2 in spleen). However, liver of these animals exhibited normal architecture (score: 1). In case of immunization with Mtb*∆mms*, the animals exhibited a lung phenotype similar to BCG immunized animals (score: 2-3). However, in the case of spleen and liver, vaccination with Mtb*∆mms* resulted in an enhanced pathology when compared with the BCG vaccination (score: 2-3 in spleen and 3-4 in liver) (Figure 9A).

 Histopathological analyses of lung and liver sections further substantiated the gross pathological observations. At 4 weeks after challenge, sham immunized animals exhibited several discrete necrotic tubercles occupying 30-40% of the lung sections (Figure 8B). Vaccination with BCG or Mtb*∆mms* prevented pulmonary damage as was evident from the presence of only a moderate granulomatous infiltration and well preserved alveolar spaces, when compared with the sham immunized animals. Sham immunized animals displayed inflammation of hepatic tissues with scattered areas of cellular infiltration. BCG vaccinated animals exhibited minimal involvement of hepatic tissues; however, Mtb*∆mms* immunized guinea pigs exhibited moderate involvement with granulomatous infiltration (Figure 8B). Further, at 12 weeks after challenge, as expected, lungs of the sham immunized animals exhibited extensive granulomatous infiltration with multi-focal coalescing granulomas along with prominent central coagulative necrosis (Figure 9B). BCG immunized animals exhibited the presence of scattered areas of inflammation with discrete granulomas along with necrotic centre in lungs. Mtb∆*mms* vaccinated animals exhibited moderate inflammation in lungs similar to BCG vaccinated animals. On comparing the pathological changes in liver (Figure 9B), sham immunized animals exhibited effacement of a large proportion of hepatic parenchyma due to multiple coalescing foci of necrotic granulomas. Immunization with BCG resulted in a significant reduction in hepatic damage with only a negligible granulomatous infiltration. However, in the case of Mtb∆*mms* immunization, the hepatic lobules displayed an extensive granulomatous infiltration (Figure 9B). 

## Discussion

 The development and widespread administration of the BCG vaccine since the early 1920s was originally hailed as a major breakthrough with the promise to eradicate the scourge of TB from the world. However, the early promise was not realized and with the growing incidence of TB cases and inconsistent protective efficacy of BCG, it became evident that the BCG vaccine, in its existing form is of limited use in controlling the disease particularly in the elderly [36]. The availability of complete *M.tuberculosis* genome sequence and an increased understanding of the genes involved in *M.tuberculosis* virulence and immune responses has led to a renewed optimism that it should be possible to develop more efficient TB vaccines than the existing BCG [37,38].

In this study, we have developed a multigene mutant of *M.tuberculosis*, having deletions in three genes namely, *mptpA*, *mptpB* and *sapM* that are involved in host-pathogen interaction and signal transduction. *M.tuberculosis* Erdman and *M.tuberculosis* H37Rv have been commonly used as basis for generating attenuated strains of *M.tuberculosis* [39–44]. However, to ensure that their virulence was not diminished on account of repeated *in vitro* subculturing, the bacilli recovered from the organs of infected animals were subcultured only once for their use for generating mutants or for challenging the animals.

We have evaluated the vaccine efficacy of the resulting mutant against *M.tuberculosis* challenge in guinea pigs. To ensure that the mutated genes were essential only for the growth of the pathogen in the host and not in the broth culture, we selected the genes implicated in the host-pathogen interaction. As expected, MtbΔ*mms* grew in broth culture similar to the parental strain, however, it displayed a significantly reduced ability to infect and grow inside the human THP-1 macrophages emphasizing that the phosphatases are vital for the growth of the pathogen in macrophages.

 In addition, our studies in guinea pigs also provide evidence that MtbΔ*mms* is highly attenuated for its growth and ability to cause pathology in the host. On inoculation of guinea pigs with Mtb∆*mms*, no bacilli were recovered from the lungs of the animals at any time point of the study. From the spleens of these animals, bacilli were recovered during early phases after the inoculation (4 weeks) but no bacilli were recovered after this initial period. This demonstrated that MtbΔ*mms* could survive only for a short while; therefore, at 10 weeks post inoculation, the organs of MtbΔ*mms* as well as BCG inoculated animals appeared to be similar with no apparent damage indicating that the mutant was safe to be used as a vaccine candidate.

 For the evaluation of protective efficacy, we employed guinea pig model of experimental tuberculosis. The guinea pig model of low dose aerogenic infection with virulent *M.tuberculosis* has been preferentially used to elucidate the events in the pathogenesis of pulmonary tuberculosis [45]. Guinea pigs are more susceptible to tuberculosis infection and have the advantage over mice in that the pathology of the disease in this model is closer to human tuberculosis. Thus, it serves as an effective model to evaluate vaccine efficacy. When guinea pigs are infected with less than 10 CFU of virulent *M.tuberculosis*, it has been observed that the pathogen disseminates from lungs to the pulmonary lymph nodes via hematogenous spread and then appears in spleens within ~3 weeks post infection [46,47]. Bacilli reseed the lung by ~4 weeks to form secondary granulomas. The protective efficacy of Mtb∆*mms* was evaluated based on its ability to reduce the bacillary load in lungs and spleens of guinea pigs post *M.tuberculosis* infection as well as to control the pathological damage. 

 Our study shows that Mtb∆*mms* vaccination was able to restrict bacterial multiplication at the primary site of infection leading to reduction in the pulmonary bacillary load and this bacillary load reduction by Mtb∆*mms* as well as by BCG was comparable during early phase (4 weeks) after infection. Unlike in the case of BCG vaccination, however, Mtb∆*mms* was not able to control hematogenous spread. A number of studies have reported the examples of vaccines which fail to provide consistent protection in all the organs uniformly [12,48,49]. For example, it has been reported that a recombinant BCG expressing ESAT-6 provided significant protection in both mice and guinea pigs against dissemination at extra-pulmonary site but failed to protect against pulmonary form of the disease [49]. On the other hand, vaccination of guinea pigs with DNA encoding the mycobacterial antigen MPB83 influenced the pulmonary pathology but not the hematogenous spread following the aerogenic infection with *Mycobacterium bovis* [48]. Also, in the case of vaccination of guinea pigs with the ∆*phoP* mutant, a significant reduction in the bacillary load in lungs but not in the spleens was observed as compared to the unvaccinated animals [12]. 

 On extending the period between the *M.tuberculosis* challenge and euthanasia to 12 weeks, although BCG appeared to lose the control of bacillary multiplication in the pulmonary tissue, Mtb∆*mms* was still very effective in controlling the lung infection. At this time point, however, neither BCG nor Mtb∆*mms* exhibited any significant control over the hematogenous spread. The pathological damage in the animals from various groups corroborated the CFU data. From this, we could infer that Mtb∆*mms* imparted as much or better control of the disease than BCG at the pulmonary site. However, immunization with the phosphatase mutant did not show any superior control over the bacillary multiplication in spleens, when compared with the sham immunized animals.

As phosphatases play an important role in the lipid metabolism, we evaluated the lipid profiles of *M.tuberculosis* and MtbΔ*mms* to ascertain whether disruption of phosphatase genes might result in the altered lipid profile in MtbΔ*mms*. However, our observations demonstrated that the lipid profiles of both the strains were identical inspite of disruption of phosphatase genes in MtbΔ*mms* indicating thereby that the phenotype of the mutant was ascribed to the loss of phosphatase genes and the influence was not related to any alteration in the lipid composition. 

 To summarize, we demonstrate that mutation of genes encoding the signal transduction associated phosphatases of *M.tuberculosis* provides optimism for the generation of novel potential vaccine candidates against tuberculosis. The Mtb∆*mms* was not only significantly attenuated for growth in macrophages and guinea pigs, it also imparted an enhanced protection against pulmonary TB. However, further modifications would be required in order for Mtb∆*mms* to elicit more appropriate immune responses for imparting superior protection including the control of hematogenous spread. Moreover, due to increasing concern about the emergence of antibiotic resistance in human pathogens, use of antibiotic-resistant genes in recombinant vaccines meant for use in humans is not permissible [50]. Hence, the antibiotic resistance genes from MtbΔ*mms* would have to be removed before any possibility of its use in human clinical trials. Our future efforts would focus on addressing these issues.

## Supporting Information

Table S1 **Solvent systems employed for lipid analyses.**
(DOC)
