# Expression of Concern: Secretory Phosphatases Deficient Mutant of *Mycobacterium tuberculosis* Imparts Protection at the Primary Site of Infection in Guinea Pigs

**DOI:** 10.1371/journal.pone.0277782

**Published:** 2022-11-10

**Authors:** The PLOS ONE Editors

After this article [1] was published, errors came to light involving Figs 1, 2, 5, 8 and 9:

- There are undeclared image splice lines in Figs 1F and 2B.
- The whole organ images of the BCG immunized liver and spleen in Fig 5 were duplicated as representing liver and spleen of the BCG immunized group in Fig 8.
- The whole organ image of the BCG immunized spleen in Fig 9 was duplicated as representing the spleen in the MtbΔ*mms* immunized group in Fig 8.

**Fig 8:**
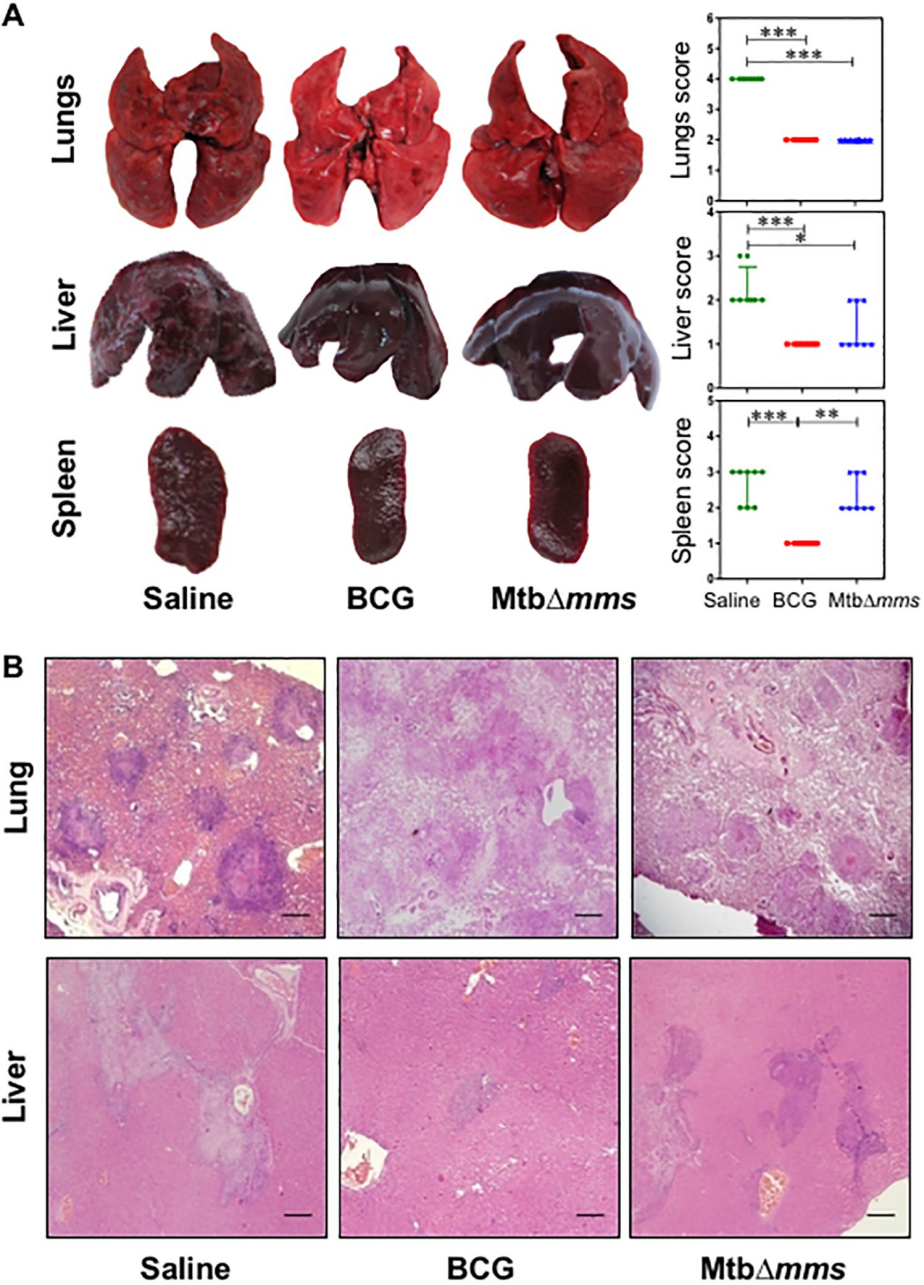
Gross pathology and histopathology of vaccinated guinea pig organs at 4 weeks after challenge. (A) Representative photographs of lungs, liver and spleen of individual animal (n = 8) vaccinated with either BCG, MtbΔ*mms* or saline and euthanized at 4 weeks after challenge. The graphical representation of gross scores of various organs is also shown alongside. The bar depicts median for each group. *p<0.05; **p<0.01 and ***p<0.001 (Kruskal Wallis test). (B) The representative photomicrographs of the lung and liver tissues stained with Haematoxylin-Eosin staining (H&E). The figure depicts 20x magnification of representative photomicrographs of organs of the animals vaccinated with either BCG, MtbΔ*mms* or saline and euthanized at 4 weeks after challenge. The scale bars depict 500 μm for lung as well as liver sections. Unvaccinated animals show severe pathology in lungs characterized by the presence of numerous large and small sized tubercles. However, hepatic and splenic tissues show moderate involvement. In the case of immunization with MtbΔ*mms*, the animals show moderately inflamed lungs with minimal hepatic tissue destruction similar to that observed in the case of BCG vaccination. However, spleens of MtbΔ*mms* vaccinated animals exhibit pathological damage similar to sham immunized animals.

The authors provided image data for Figs 1F and 2B (S1 File) which clarified that areas between the lanes of interest were removed in preparing these figures.

The authors also noted that the whole organ image duplications arose due to errors in preparing Fig 8. They apologized for the errors and provided a corrected figure as well as the original images underlying Figs 5, 8 and 9 (S1 File). They stated that the errors did not affect the quantitative data shown in the Figs 5, 8 and 9.

A member of *PLOS ONE*’s Editorial Board reviewed the updated figure and the underlying data provided. The Academic Editor advised that further pathological and histological analyses and disease readouts are needed to clarify the nature of the lesions observed *in vivo* and to support claims made in the article about organ structure and pathology. The corresponding author disagreed and commented that the methods used in the study are suitable for evaluating TB disease pathogenesis.

The following issues were also identified in the post-publication assessment:

- Claims about lack of infection (e.g. “No bacilli were detectable…”) are based on bacillary load data for partial organs and may not be representative of whole organ bacillary loads.
- Claims about whether the mutant could be safely used as a vaccine candidate are not adequately supported; additional safety & toxicity assessments would be needed to support such claims. This issue calls into question the reliability of the following statements:
  - “…it appeared that as a result of deletion of the phosphatase genes, MtbΔ*mms* was sufficiently attenuated for growth in the host tissues and could be safely used as a vaccine candidate.”
  - “the MtbΔ*mms* mutant appeared to be safe for its use as a vaccine candidate”
  - “…at 10 weeks post inoculation, the organs of MtbΔ*mms* as well as BCG inoculated animals appeared to be similar with no apparent damage indicating that the mutant was safe to be used as a vaccine candidate.”
  The corresponding author agreed that additional safety and toxicity assessment studies are needed for further development of the mutant strain as an auxotrophic vaccine. He clarified that this particular study was carried out with the idea of providing a proof of concept and exploring a potential vaccine candidate, to be worked upon further if it showed promise.
- The following subtitle in the Results section overstates what can be drawn from the reported data, and is misleading in light of pathology results reported for the 4 week timepoint: “Deletion of phosphatase genes renders *M*. *tuberculosis* incapable of causing pathology in guinea pigs at 10 weeks post inoculation”.
- The Results paragraph discussing the Fig 7D experiment include the statement, “These observations demonstrated that MtbΔ*mms* exhibited a more sustainable and superior protection as compared to BCG.” Similarly, the conclusions state that the MtbΔ*mms* mutant “imparted an enhanced protection against pulmonary TB” [1]; the implication is that this refers to enhanced protection vs. BCG. These statements in the Results and Conclusions are not supported by the data: no statistically significant difference between MtbΔ*mms* vs. BCG groups was observed according to results reported in Figs 7 and 9. At the 12 week timepoint, a significant difference in pulmonary bacillary load was observed for MtbΔ*mms* vs. saline control groups but not for BCG vs. saline control groups. This suggests that MtbΔ*mms* may provide a more sustained protective response than BCG against pulmonary TB infection, but further studies are needed to explore this hypothesis.

Based on the outcome of our post-publication assessment, the *PLOS ONE* Editors concluded that the results in Figs 3, 4 and 7 support the article’s main claims about the MtbΔ*mms* mutant’s attenuated growth in macrophages and guinea pigs and ability to impart protection against pulmonary TB in guinea pigs. However, the article’s claims about organ pathology, the safety of MtbΔ*mms* for use as a possible vaccine candidate, and enhanced protection by MtbΔ*mms* (vs. BCG) are not adequately supported by the reported experiments and data.

The conclusions are hereby updated as follows:

- **Original**: “The MtbΔ*mms* was not only significantly attenuated for growth in macrophages and guinea pigs, it also imparted an enhanced protection against pulmonary TB.” [1]
- **Revised**: “The MtbΔ*mms* mutant was significantly attenuated for growth in macrophages and guinea pigs, and appeared to impart protection against pulmonary TB infection as evidenced by bacillary load data and whole organ assessments in a guinea pig model. However, spleen data from the in vivo model indicated that the MtbΔ*mms* mutant is less effective than BCG at controlling hematogenous spread. Further studies are needed to (a) explore the safety and effectiveness of Mtb mutants as potential vaccine candidates, using additional histopathological analyses, toxicity assessments, and disease readouts, and (b) identify and investigate potential candidates that provide clinical advantages over BCG considering whole animal outcomes with regard to infection, pathology, safety and toxicity, and disease progression.”

The *PLOS ONE* Editors issue this Expression of Concern to notify readers of the above issues and the revised Conclusions.

## Supporting information

S1 File Underlying data for Figs 1F, 2B, 5A, 8A and 9A. (ZIP)
