# Supplementary material for: Secretory Phosphatases Deficient Mutant of Mycobacterium tuberculosis Imparts Protection at the Primary Site of Infection in Guinea Pigs
Source: PLoS One. 2013 Oct 18;8(10):e77930. doi: 10.1371/journal.pone.0077930 (PMC3799640; doi:10.1371/journal.pone.0077930)
Supplement: Table S1 — Solvent systems employed for lipid analyses. (DOC) [file pone.0077930.s001.doc]

**Table S1. Solvent systems employed for lipid analyses.**

| **Solvent System** | **1^st^ Direction** | **2^nd^ Direction** | **Fraction** | **Lipids** |
| --- | --- | --- | --- | --- |
| A | Hexane: ethylacetate (95:5 v/v) run seven times |  | - | Mycolic acids |
| B | Petroleum ether: diethyl ether (90:10 v/v) |  | Apolar | PDIM, TAG |
| C | Chloroform: methanol:water  (60:30:6 v/v/v) | Chloroform:acetic acid: methanol:water  (40:25:3:6 v/v/v/v) | Polar | PIM, PI, P |
| D | Chloroform: methanol (96:4 v/v) | Toluene : acetone (80:20 v/v) | Apolar | DAG, free fatty acids, free mycolic acids |
| E | Chloroform: methanol:water  (100:14:0.8 v/v/v) | Chloroform:  acetone:methanol:water  (50:60:2.3:3 v/v/v/v) | Apolar | GMM, SLIII, DAT, TMM, TDM |

PDIM- phthiocerol dimycocerosate, TAG- triacylglycerol, DAG- diacylglycerol, GMM- glucose monomycolate, SL- sulfolipids, DAT- diacyltrehalose, TMM- trehalose monomycolate, TDM- trehalose dimycolate, PIM- phosphatidylinositol mannoside, PI- phosphatidylinositol, P- phospholipids.
