# Supplementary material for: Expression of Concern: Secretory Phosphatases Deficient Mutant of Mycobacterium tuberculosis Imparts Protection at the Primary Site of Infection in Guinea Pigs
Source: PLoS One. 2022 Nov 10;17(11):e0277782. doi: 10.1371/journal.pone.0277782 (PMC9648787; doi:10.1371/journal.pone.0277782)

Mtb infected animals – Figure 5

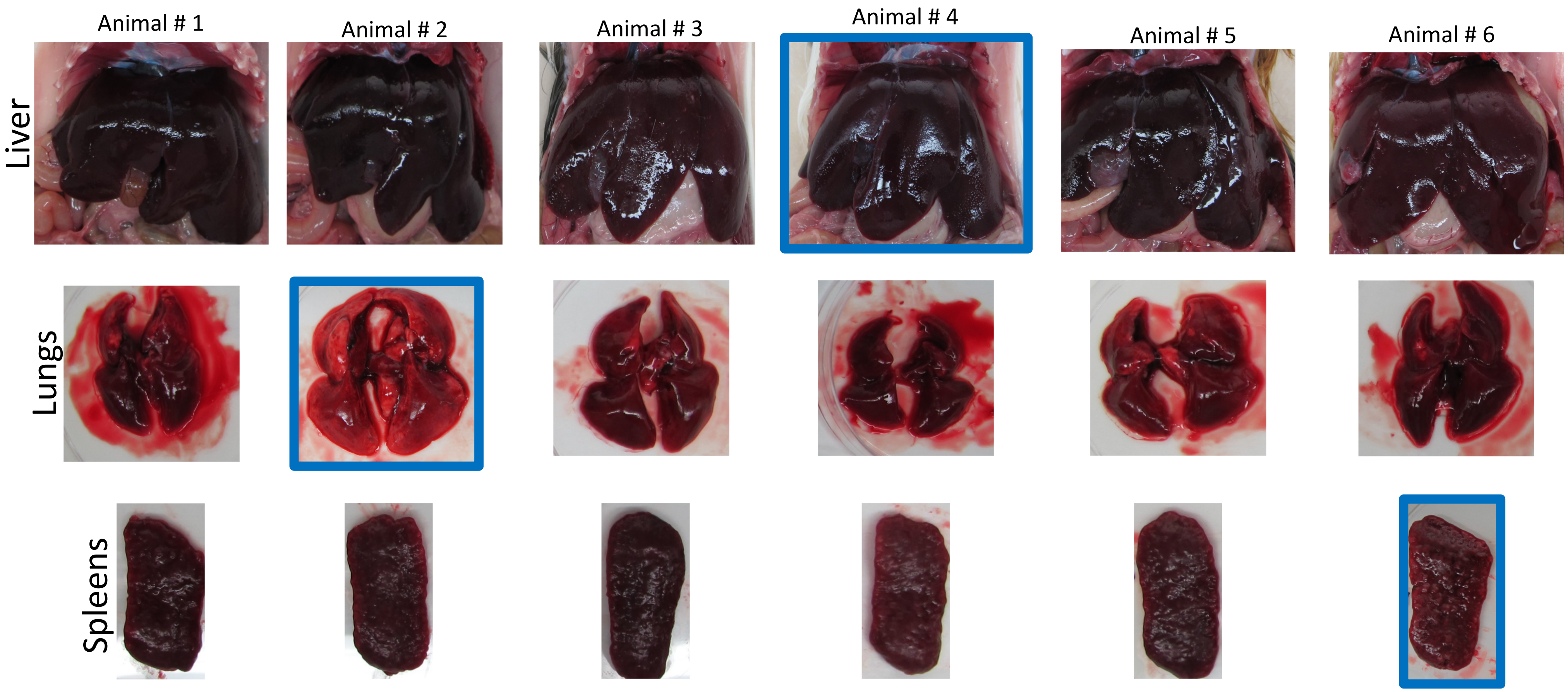

BCG infected animals – Figure 5

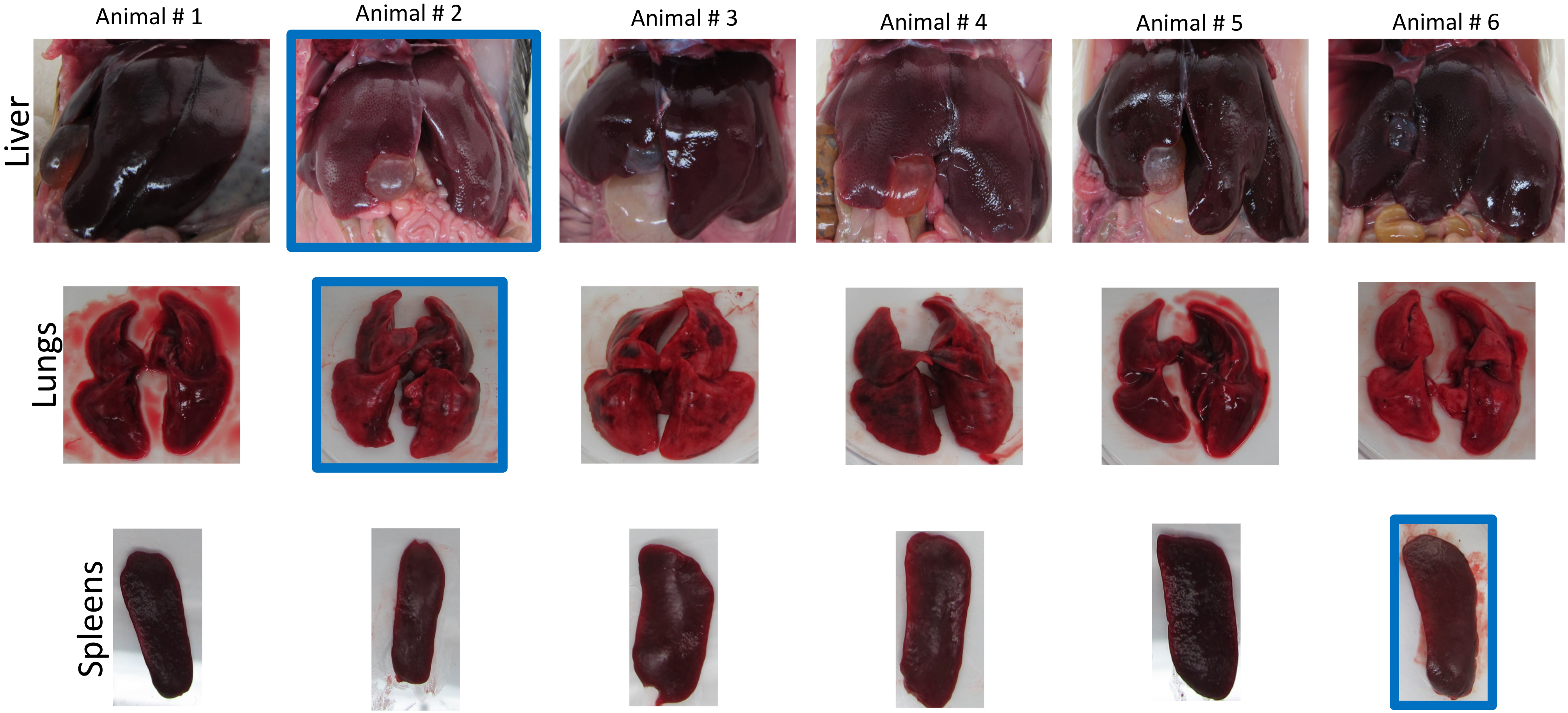

Δmms infected animals – Figure 5

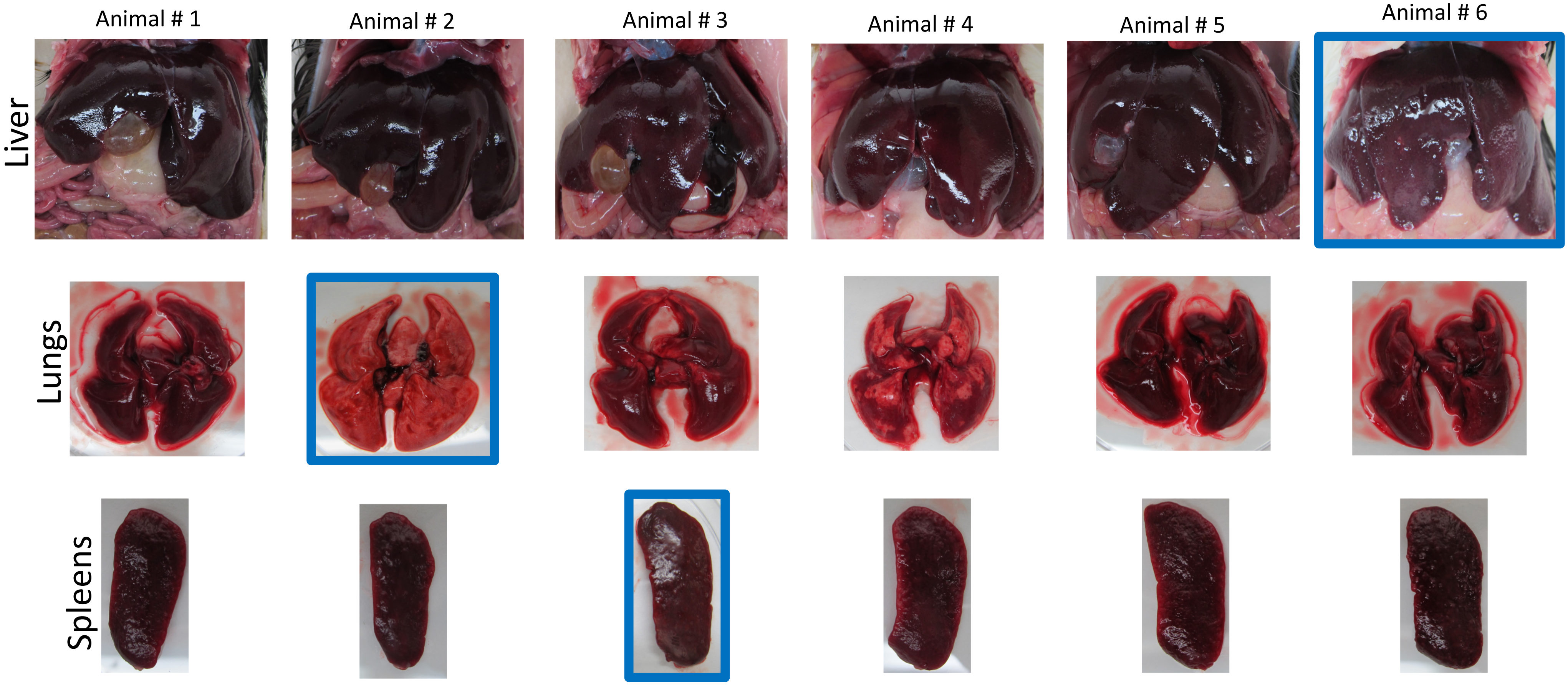

Supplement: S1 File — (ZIP) [file pone.0277782.s001.zip › Figure 5A.pdf]
