# Supplementary material for: Expression of Concern: Secretory Phosphatases Deficient Mutant of Mycobacterium tuberculosis Imparts Protection at the Primary Site of Infection in Guinea Pigs
Source: PLoS One. 2022 Nov 10;17(11):e0277782. doi: 10.1371/journal.pone.0277782 (PMC9648787; doi:10.1371/journal.pone.0277782)
Supplement: S1 File — (ZIP) [file pone.0277782.s001.zip › File S6.pdf]

Fig. 5A Scoring data

Lung scoring data			
Animal No.	Mtb	BCG	mms
1	2	1	1
2	2	1	1
3	2	2	1
4	2	1	2
5	2	1	1
6	2	1	1

Liver scoring data			
Animal No.	Mtb	BCG	mms
1	2	1	1
2	2	1	1
3	2	1	1
4	1	1	1
5	2	1	1
6	2	1	2

Spleen scoring data			
Animal No.	Mtb	BCG	mms
1	3	1	2
2	3	1	2
3	3	1	2
4	2	1	2
5	3	1	2
6	3	1	3

Fig. 8A Scoring data

Lung scoring data			
Animal No.	Saline	BCG	mms
1	4	2	2
2	4	2	2
3	4	2	2
4	4	2	2
5	4	2	2
6	4	2	2
7	4	2	2
8	4	2	2

Liver scoring data			
Animal No.	Saline	BCG	mms
1	3	1	1
2	2	1	1
3	3	1	1
4	2	1	2
5	2	1	2
6	2	1	2
7	2	1	1
8	2	1	1

Spleen scoring data			
Animal No.	Saline	BCG	mms
1	3	1	3
2	3	1	2
3	2	1	3
4	3	1	3
5	2	1	2
6	2	1	2
7	3	1	2
8	3	1	2

Fig. 9A Scoring data

Lung scoring data			
Animal No.	Saline	BCG	mms
1	4	2	2
2	4	3	2
3	4	3	1
4	3	1	2
5	3	2	4
6	4	3	2
7	2	3	2
8	4	3	3

Liver scoring data			
Animal No.	Saline	BCG	mms
1	4	1	4
2	3	1	4
3	4	1	1
4	4	1	3
5	3	1	4
6	1	1	4
7	4	1	3
8	4	1	4

Spleen scoring data			
Animal No.	Saline	BCG	mms
1	3	2	3
2	4	2	3
3	4	2	2
4	4	1	2
5	4	2	3
6	4	2	3
7	2	1	3
8	1	1	3
