# Supplementary figures and images for: Expression of Concern: Secretory Phosphatases Deficient Mutant of Mycobacterium tuberculosis Imparts Protection at the Primary Site of Infection in Guinea Pigs
Source: PLoS One. 2022 Nov 10;17(11):e0277782. doi: 10.1371/journal.pone.0277782 (PMC9648787; doi:10.1371/journal.pone.0277782)

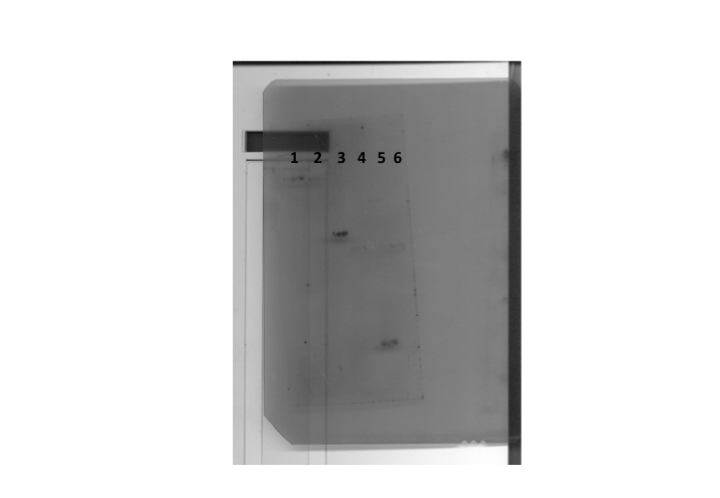

Supplement: S1 File — (ZIP) [file pone.0277782.s001.zip › Fig 1F.tiff]

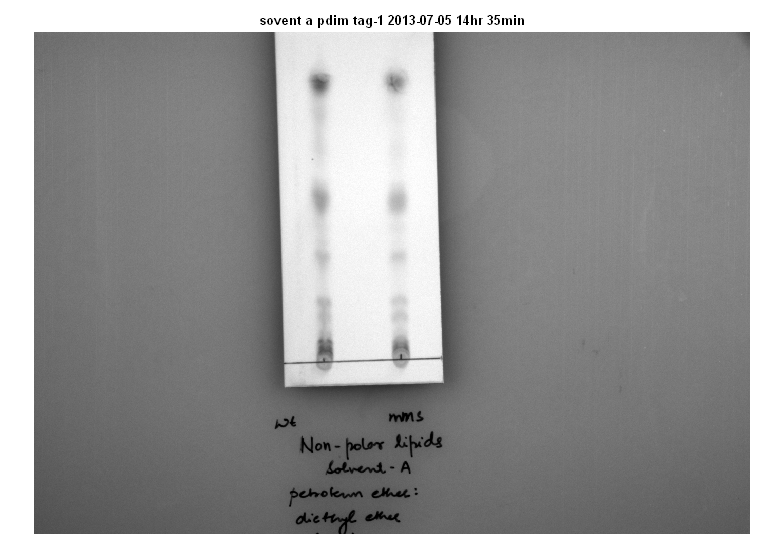

Supplement: S1 File — (ZIP) [file pone.0277782.s001.zip › Fig 2B.tiff]

# Sham immunized animals – Figure 8

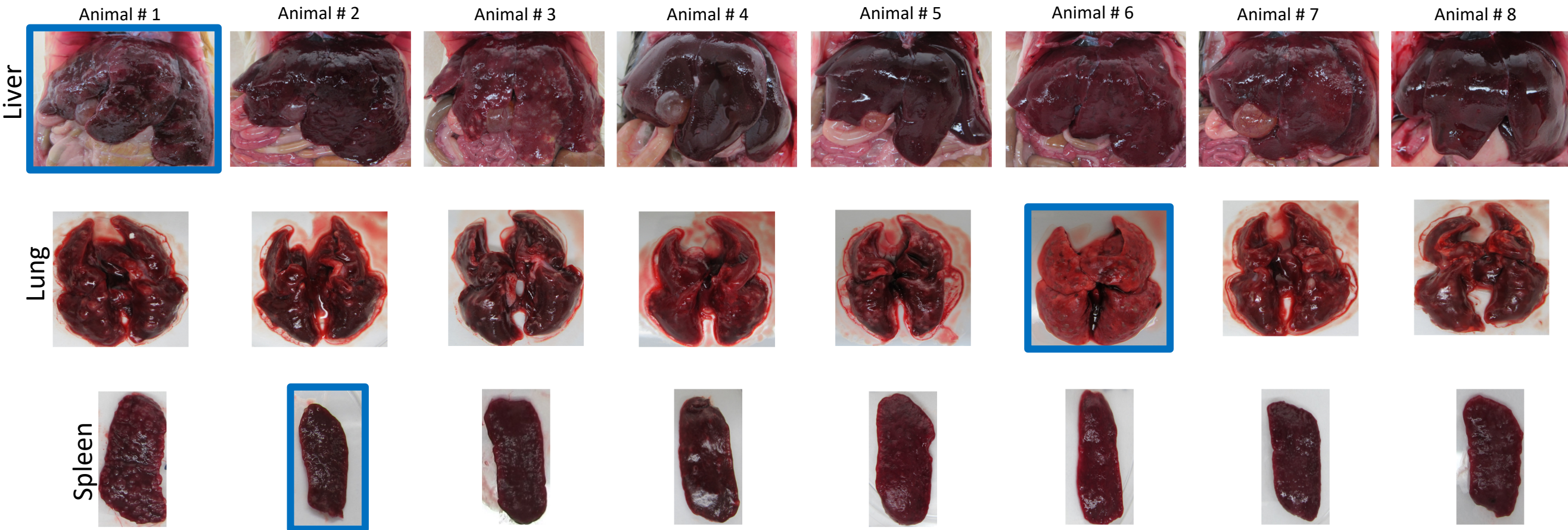

# BCG immunized animals – Figure 8

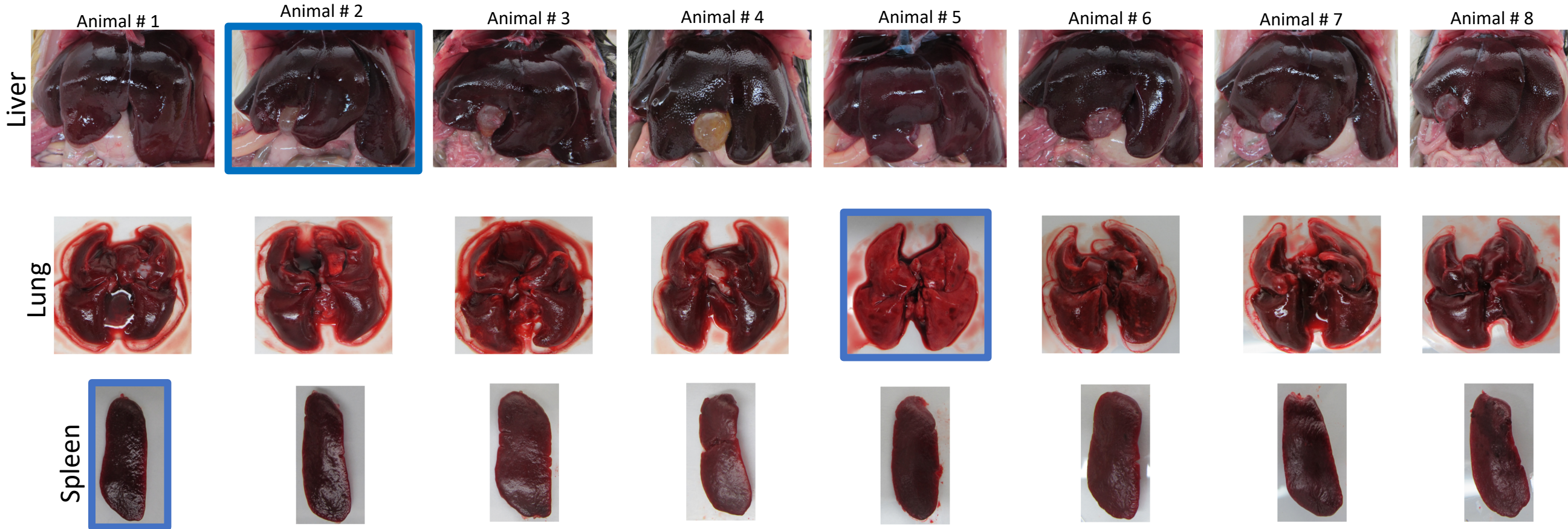

# mms immunized animals – Figure 8

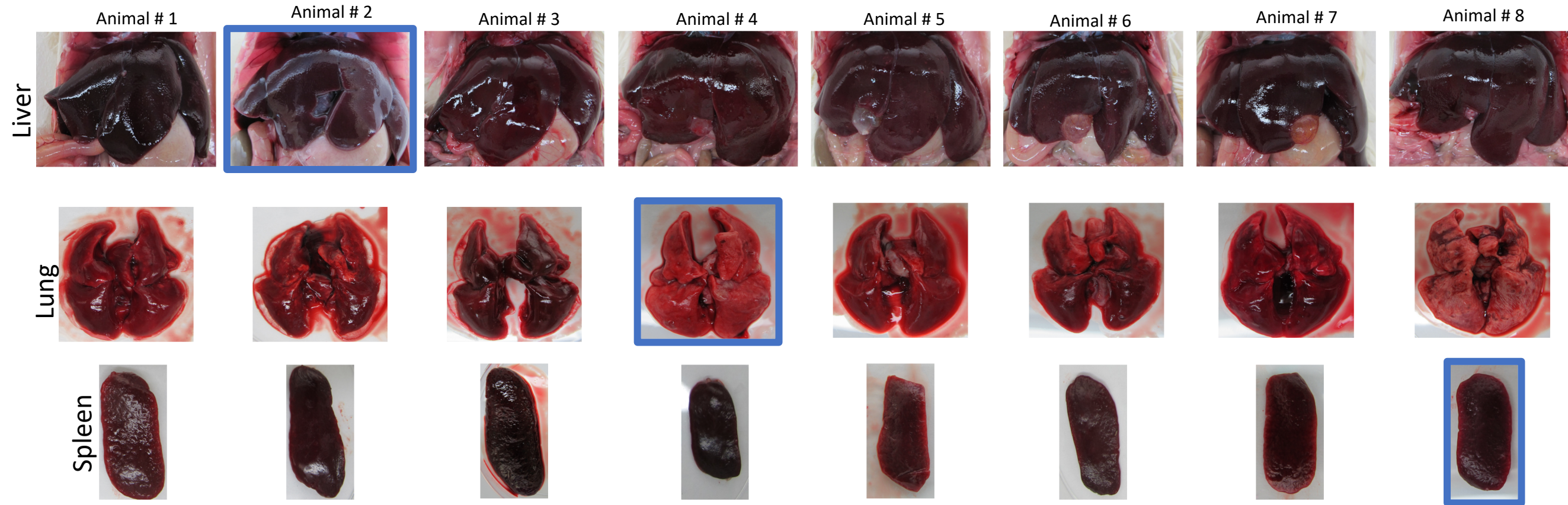

Supplement: S1 File — (ZIP) [file pone.0277782.s001.zip › Figure 8A.pdf]
